# A distinctive distribution of hypoxia‐inducible factor‐1α in cultured renal tubular cells with hypoperfusion simulated by coverslip placement

**DOI:** 10.14814/phy2.14689

**Published:** 2020-12-28

**Authors:** Tomoko Honda, Yosuke Hirakawa, Kiichi Mizukami, Toshitada Yoshihara, Tetsuhiro Tanaka, Seiji Tobita, Masaomi Nangaku

**Affiliations:** ^1^ Division of Nephrology and Endocrinology Graduate School of Medicine The University of Tokyo Tokyo Japan; ^2^ Graduate School of Science and Technology Gunma University Gunma Japan

**Keywords:** hypoperfusion, hypoxia, hypoxia‐inducible factor, oxygen gradient, pH

## Abstract

Chronic hypoxia in the renal tubulointerstitium plays a key role in the progression of chronic kidney disease (CKD). It is therefore important to investigate tubular hypoxia and the activity of hypoxia‐inducible factor (HIF)‐1α in response to hypoxia. Rarefaction of the peritubular capillary causes hypoperfusion in CKD; however, the effect of hypoperfusion on HIFs has rarely been investigated. We induced hypoperfusion caused by coverslip placement in human kidney‐2 cells, and observed an oxygen gradient under the coverslip. Immunocytochemistry of HIF‐1α showed a doughnut‐shaped formation on the edge of a pimonidazole‐positive area, which we named the “HIF‐ring”. The oxygen tension of the HIF‐ring was estimated to be between approximately 4 mmHg and 20 mmHg. This result was not compatible with those of past research showing HIF‐1α accumulation in the anoxic range with homogeneous oxygen tension. We further observed the presence of a pH gradient under a coverslip, as well as a shift of the HIF ring due to changes in the pH of the culture medium, suggesting that the HIF ring was formed by suppression of HIF‐1α related to low pH. This research demonstrated that HIF‐1α activation mimics the physiological state in cultured cells with hypoperfusion.

## Graphical abstract

We examined HIF‐1α distribution in the hypoperfusion model using a coverslip and immunicytochemistry of HIF‐1α in HK‐2 cells showed a doughnut‐shaped enhancement just on the edge of the pimonidazole‐positive area, which we called the “HIF ring”. We discovered that there was pH gradient as well as oxygen gradient at the coverslip edge in the hypoperfusion model, induced by coverslip placement. We proposed the possible mechanism of HIF ring formation that, despite HIF‐1α accumulation with decreasing oxygen tension, the accumulation was suppressed because of low pH within a certain distance from a coverslip edge.

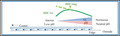

## 1 INTRODUCTION

The incidence of chronic kidney disease (CKD) is increasing across the world as societies age (Tonelli & Riella, 2014). The progression of CKD is irreversible once renal damage reaches a certain degree, and finally results in end‐stage renal disease (ESRD), regardless of underlying disease. This outcome indicates the existence of a “final common pathway.” According to past pathological analyses, decline in renal function is more strongly correlated with tubulointerstitial damage than with glomerular damage. There have been several reports demonstrating that renal fibrosis induces chronic hypoxia in the tubulointerstitium, and this tubulointerstitial hypoxia aggravates CKD and leads to ESRD. This evidence indicates that tubulointerstitial hypoxia plays a part in the final common pathway of CKD (Mimura & Nangaku, 2010; Nangaku, 2006). The kidney is highly susceptible to hypoxia because of its high demand for oxygen, and the existence of an oxygen shunt between the intrarenal arteries and veins (Nangaku, 2006; Welch et al., 2001; Zhang et al., 2014). Therefore, we consider that it is essential to investigate hypoxia and the response to hypoxia in the renal tubulointerstitium.

The primary biological response to hypoxia in living organisms occurs via the hypoxia‐inducible factor (HIF) pathway (Hypoxia, 2011; Semenza & Wang, 1992; Zhou et al., 2003). HIF consists of α‐ and β‐subunits. Although the β‐subunit is constitutively active, the α‐subunit is degraded in the presence of oxygen. Under normoxic conditions, HIF‐α is hydroxylated by the prolyl hydroxylase domain (PHD), making it recognizable by the von Hippel–Lindau tumor suppressor (VHL). This recognition results in ubiquitination and degradation of hydroxylated HIF‐α in the proteasome. Under hypoxic conditions, HIF‐α accumulates in the cytosol, because non‐hydroxylated HIF‐α escapes degradation. Accumulated HIF‐α is translocated from the cytosol to the nucleus, where it dimerizes with HIF‐β, and acts as a transcriptional factor, promoting the expression of downstream genes. Of the three identified isoforms of HIF‐α subunits—HIF‐1α, HIF‐2α, and HIF‐3α—renal tubular epithelial cells are known to express HIF‐1α (Tanaka et al., 2016). Previous studies have shown transient or regional accumulation of HIF in the kidney with CKD (Goldfarb et al., 2006; Yu et al., 2012), and this HIF is activated in the reduced oxygen tension in the CKD milieu. The maladaptation to hypoxia in CKD might be caused by the presence of factors that suppress HIF pathways (Asai et al., 2016; Tanaka et al., 2013; Thangarajah et al., 2009). Protective effects of HIF activation have been reported in several animal models, including streptozotocin‐induced diabetic rats and 5/6th nephrectomy rats (Deng et al., 2010; Nordquist et al., 2015). PHD inhibitors have recently attracted attention as novel therapeutic approaches to renal anemia in patients with CKD (Akizawa et al., 2019; Chen et al., 2017, 2019; Coyne et al., 2017; Miyata et al., 2011; Pergola et al., 2016; Provenzano et al., 2016). However, the details of the progression of renal hypoxia, and the way in which HIF accumulation occurs in the kidney with CKD, remains obscure.

Oxygen‐dependent hydroxylation of HIF‐α occurs in the cytosol, and it is therefore important to measure intracellular and extracellular oxygen tension. There are several methods used for measuring oxygen tension, including the use of microelectrodes or blood oxygen level‐dependent magnetic resonance imaging (BOLD‐MRI). We used phosphorescence lifetime imaging microscopy (PLIM) for quantitative measurement of the intracellular oxygen status (Hirakawa et al., 2017; Yoshihara et al., 2015). BTPDM1 is a phosphorescent dye based on the iridium (III) complex BTP, (btp)_2_Ir(acac) (btp = benzothienylpyridine, acac = acetylacetone) with a cationic dimethylamino group, which is passively distributed in intracellular lysosomes. It was synthesized as a phosphorescent probe to measure the intracellular oxygen pressure (Yoshihara et al., 2015). PLIM with BTPDM1 enabled the acquisition of high‐resolution images of the partial pressure of oxygen in renal tubular cells on the renal surface of normal mice, providing data indicating the presence of an oxygen gradient, even in normal kidneys (Hirakawa et al., 2018).

There are oxygen gradients in living organs, including kidneys, even under normal conditions (Hirakawa et al., 2018; Kietzmann, 2017; Zhdanov et al., 2015), and such gradients can therefore also be expected in CKD. Hypoperfusion is caused by rarefaction of the microvasculature in CKD, in which progressive glomerular injury results in a decrease in peritubular capillary blood flow, aggravating interstitial fibrosis. Interstitial fibrosis impairs the diffusion and supply of oxygen to the tubular cells, and leads to rarefaction of the microvasculature, further aggravating tubulointerstitial hypoxia (Mimura & Nangaku, 2010; Nangaku, 2006). The biological effects of hypoperfusion, however, remain obscure, because most studies have investigated the effects of hypoxia and HIF‐1α on cultured cells under conditions of homogenous perfusion and oxygen tension (Rexius‐Hall et al., 2017). Previous studies have demonstrated that monolayered cultured cells covered with a coverslip provide a good model of hypoperfusion induced by a barrier to diffusion in the culture medium. The modeled hypoperfusion is associated with an oxygen gradient because a coverslip prevents oxygen diffusion from the top of the medium to the cells (Pitts & Toombs, 2004; Takahashi & Sato, 2010; Yoshihara et al., 2015). In a monolayer of cultured cells covered with a coverslip intracellular oxygen tension drops with distance from the edge of the coverslip because of limited oxygen diffusion into cultured cells. As a result, an oxygen gradient is formed from the edge to the center of the coverslip, and the intracellular oxygen tension may drop to an anoxic range in the center of the coverslip (Takahashi & Sato, 2010; Yoshihara et al., 2015). In the current study, we focused on HIF activation, and investigated whether there is an oxygen‐independent change in HIF‐α expression in the presence of hypoperfusion with an oxygen gradient. Since hypoperfusion with an oxygen gradient exists in renal tubules *in vivo*, the observation of oxygen‐independent effects on HIF‐α expression in the coverslip model can provide insights into the mechanism underlying insufficiency of HIF‐α accumulation in CKD.

## 2 MATERIALS AND METHODS

### 2.1 Cell culture

Cultured cells were incubated in a humidified incubator with 5% CO_2_. Hypoxic incubation was performed in a personal CO_2_/multi gas incubator APM‐30D (Astec). Anoxia was induced with an anoxia bag, AnaeroPack, and Anaerobic cultivation sets #A‐13 (Mitsubishi Gas Chemical).

HK‐2 cells (*Homo sapiens*, human kidney, male, CRL‐2190, ATCC, RRID: CVCL_0302), an immortalized proximal tubule epithelial cell line from normal adult human kidney, were cultured in Dulbecco's modified Eagle's medium/nutrient mixture F‐12 Ham (DMEM/F12) (D8062, Sigma Aldrich) containing 10% fetal bovine serum (FBS) (F7524, Sigma Aldrich), and penicillin‐streptomycin solution (15070063, Thermo Fisher Scientific) in a 10 cm culture dish. For passage, HK‐2 cells were dissociated with trypsin (204‐16935, Wako Pure Chemical Industries, Ltd.) and centrifuged at 300 *g* for 5 min.

HeLa cervical cancer cells and human embryonic kidney cells 293 (HEK293) were cultured in DMEM with low (1000 mg/L) glucose (D6046, Sigma Aldrich) containing 5% FBS in a 10 cm culture dish. These cells were passaged as HK‐2 cells.

Renal proximal tubule epithelial cells (RPTECs) (CC‐2553, Cambrex) were maintained using RenaLife^TM^ Comp kits (LRC‐LL0025, Lonza Ltd.). For passage, RPTECs were dissociated using trypsin, neutralized with Trypsin Neutralizing Solution (CC‐5002, Lonza Ltd.) and centrifuged at 200 *g* for 5 min.

### 2.2 Establishment of a hypoperfusion model by coverslip placement

Round‐shaped coverslips of 15 mm diameter (C015001, Matsunami) were cleaned using ultrasound and stored in 99.5% ethanol before use. Two methods were employed, based on the observation of cells outside the coverslip.

Monolayered cultured cells were sandwiched between a coverslip and the bottom of a dish (Figure S1a,b), or an alternative method (Figure S1c), as described below. Either the traditional or the alternative method was selected to establish our coverslip model for live imaging, whether oxygen imaging or PH imaging. For immunocytochemistry (ICC), the alternative method was chosen, because when using the traditional method, the majority of cells frequently detached from the bottom of the dish during coverslip removal for cell fixation (Figure S2a).

#### 2.2.1 Traditional method

On Day 1, cells were placed on the bottom of a 27 mm glass‐based dish (3910‐035, Iwaki) at a confluence of 100%* (approximately 5.0 × 10^5^/dish). They were cultured in DMEM/F12 containing 10% FBS without antibiotic solution overnight. On Day 2, a coverslip was placed over the cultured cells for the period indicated (Figure S1b).

#### 2.2.2 Alternative method

Cells were seeded on a coverslip in a 35 mm‐culture dish at 100% confluence (approximately 5.0 × 10^5^/dish) on Day 1. They were cultured in DMEM/F12 containing 10% FBS without antibiotic solution overnight. On Day 2, the coverslip was inverted, to attach the surface of its cells to the bottom of a new 27 mm glass‐based dish for a specified period (Figure S1c).

We also created a coverslip model using round‐shaped coverslips with a diameter of 10 mm (CS01005, Matsunami) (Figure S2b).

### 2.3 Live oxygen imaging with BTPDM1

Cultured cells were prepared on Day 1, as described above. On Day 2, the cells were rinsed twice with Hanks' Balanced Salt solution (HBSS) (H8264, Sigma Aldrich) and incubated with 500 nM BTPDM1, an iridium‐based cationic lipophilic dye used as an intracellular phosphorescent probe (Yoshihara et al., 2015), in DMEM/F12 without phenol red (21041‐025, Thermo Fisher Scientific) for 30 min. After the cells were washed twice with HBSS, they were sandwiched between a coverslip and the bottom of a dish, as described above. The intensity of phosphorescence from BTPDM1 in cultured cells covered with a coverslip was observed using an excitation and emission filter, and BTPDM1 phosphorescence (Hirakawa et al., 2015) was detected using a fluorescence microscope, BZ‐X710 (Keyence Corporation). Images obtained from the inverted fluorescent microscope were adjusted for brightness and contrast using the BZ‐X analysis software.

### 2.4 Immunocytochemistry

Cells on a coverslip were cultured with 200 µM pimonidazole HCl (HP3‐100, Hypoxyprobe, Inc.) in DMEM/F12 without phenol red for 1 h, after which the coverslip was turned and attached to a cover glass dish, as described previously. The cells were then cultured in DMEM/F12 without phenol red for a specified period. When pimonidazole counterstaining was not used, pretreatment with pimonidazole was omitted.

After completion of the culture period, each coverslip was collected, and the cells were immediately fixed with methanol/acetone (1:1) on ice, where they remained for 30 min. After washing twice with Dulbecco's phosphate buffered saline (PBS) (D5652, Sigma Aldrich), the cell membrane was permeabilized for 30 min, incubated with 5% bovine serum albumin (BSA) (A3059, Sigma Aldrich) for 30 min, and subsequently with serum‐free protein block (X0909, DAKO) for 10 min. The cells were stained with a first antibody and subsequently with a second, fluorescent antibody. The list of the first antibodies is given in Table S1. FITC‐swine polyclonal anti‐rabbit immunoglobulin (F0205, 1:20 dilution, DAKO) was used as the first antibody if the host was a rabbit. The fluorescent antibody Alexa Fluor 594 streptavidin (S11227, 1:500 dilution, Thermo Fisher Scientific) was used as the first antibody if the host was a mouse, followed by biotinylated anti‐mouse IgG (H + L) (BA‐2001, 1:1000 dilution, Vector Laboratories), as the second antibody. Nuclear staining was performed using bisBenzimide H 33342 trihydrochloride (B2261, Sigma Aldrich) for each sample.

Fluorescent signals were observed using an inverted fluorescent microscope, BZ‐X710 (Keyence Corporation) with the following filters: Texas Red with an excitation wavelength (Ex) of 560/40 nm, emission wavelength (Em) of 630/75 nm, GFP (Ex: 470/40 nm, Em: 525/50 nm), and DAPI (Ex: 360/40 nm, Em: 460/50 nm). Images were adjusted for brightness and contrast using BZ‐X analysis software.

### 2.5 ICC of HIF of HK‐2 cells treated with cobalt chloride

The alternative method was modified to produce a coverslip model of HK‐2 cells treated with cobalt chloride. HK‐2 cells were seeded at half‐confluent density (2.5 × 10^5^/dish) on a coverslip on Day 1, and treated with 300 µM cobalt chloride hexahydrate (C 8661, Sigma Aldrich) for 16 h on Day 2. On Day 3, the coverslip was inverted to attach the cell surfaces to the bottom of a new 27 mm glass‐based dish for 3 h and the ICC of HIF was performed.

### 2.6 Western blotting

To investigate HIF‐1α accumulation under different oxygen tensions and at different pH levels, 1.0 × 10^6^ HK‐2 cells per 10 cm culture dish were incubated under either normoxia, 2% oxygen, 1% oxygen, or anoxia, for 5 h, or in DMEM/F12 at pH 7.4, pH 6.0, or pH 5.0 for 5 h.

These cells were lysed in RIPA buffer containing 50 mM Tris‐buffer (pH 8.0), 150 mM NaCl, 0.5 w/v% sodium deoxycholate, 0.1 w/v% SDS, and 1.0 w/v% NP40.

For western blotting, SDS sample buffer containing 0.35 M Tris‐HCl (pH 6.8), 10% SDS, 36% glycerol, 0.012% bromophenol blue, and 0.1 M dithiothreitol (DTT) was added to the proteins. Proteins containing SDS sample buffer were eluted by boiling at 95°C for 5 min.

The proteins were separated by electrophoresis on 10% SDS polyacrylamide gels. The proteins were then transferred onto an Amersham^TM^ Hybond^TM^ PVDF membrane (10600023, GE Healthcare) in transfer buffer (48 mM Tris‐base buffer, 39 mM glycine, 0.04% SDS, and 20 v/v% methanol) using a Trans‐Blot® Turbo^TM^ Transfer System (Bio‐Rad). The membranes were incubated at room temperature with primary antibodies, anti‐HIF1 α antibody (NB100‐134, 1:500 dilution, Novus Biologicals, RRID:AB_350071), and anti‐actin antibody (A2066, 1:2000 dilution, Sigma Aldrich, RRID:AB_476693), and subsequently in the secondary antibody, polyclonal goat anti‐rabbit immunoglobulin/HRP (P0448, 1:10000 dilution, DAKO, RRID:AB_2617138). Pierce^TM^ ECL Plus Western Blotting Substrate (32132, ThermoFisher Scientific) was used for detection. Chemiluminescence was observed using a Luminoimage Analyzer ImageQuantLAS4000mini (GE Healthcare). Reproducibility was confirmed by performing at least three independent experiments. The intensity of the bands was quantified using the National Institutes of Health ImageJ software (Schneider et al., 2012).

### 2.7 Luciferase reporter assay

A luciferase reporter assay was performed to measure HIF1α accumulation in homogenous hypoxic culture incubation. We previously constructed a tagged luciferase gene driven by a hypoxia‐responsive element (HRE), and developed the Hypoxia‐Responsive Reporter Vector (Transgene Construction). This was constructed from tandem copies of the HRE from the rat vascular endothelial growth factor gene sub‐cloned into the 5’ region of the hmCMV‐promoter‐luciferase transcription unit (pHRE‐luc) (Chiang et al., 2011; Tanaka et al., 2004).

HK‐2 cells at a concentration of 1.0 × 10^5^ per well were prepared in 12‐well culture plates (150628, Thermo Fisher Scientific). The cells were co‐transfected with 500 ng of pGL3‐Basic HRE‐luciferase vectors and 30 ng of pRL‐SV40 Renilla luciferase control vectors (Promega), using 2 µl FuGENE® HD Transfection Reagent (E2311, Promega) per well. HK‐2 cells co‐transfected with 500 ng of pGL3‐Basic firefly luciferase vectors and 30 ng of pRL‐SV40 Renilla luciferase control vectors (Promega) were used as a negative control.

The transfected cells were incubated under normoxia, 2% hypoxia, 1% hypoxia, or an anoxia bag, for 5 h. The cells were then harvested using 100 µl of passive protein lysis buffer, for the dual luciferase assay. An LB9507 luminometer (EG and Berthold) was used for the measurements. To correct for transfection efficiency, the relative value of the firefly luciferase light unit was divided by that of the Renilla luciferase.

### 2.8 Analysis of apoptosis

Cultured cells were covered with a coverslip for 0 min, 15 min, 30 min, 1 h, 3 h, 6 h, and 24 h, and then collected using trypsin. Cells treated with 3% hydrogen peroxide (081‐04215, Wako Pure Chemical Industries, Ltd.) for 30 min were prepared as an apoptotic control. Quantitative analysis of apoptosis was performed using Muse Annexin V and Dead Cell Kits (MCH100105, Millipore) in a Muse™ Cell Analyzer (Millipore), according to the manufacturer's instructions.

### 2.9 Quantitative real‐time PCR (qRT‐PCR)

Total RNA extraction and cDNA synthesis were carried out according to the manufacturer's instructions for RNAiso Plus (9109, Takara) and PrimeScript™ RT Master Mix (RR036B, Perfect Real Time) (Takara) respectively. Real‐time PCR was performed using THUNDERBIRD SYBR qPCR Mix (QPS‐201, Toyobo) in the CFX Connect Real‐Time PCR Detection System (Bio‐Rad). Transcript levels were normalized to the level of β‐actin mRNA expression. qRT‐PCR was performed in triplicate using gene‐specific primers. HIF‐1α was amplified using forward, 5′‐CCATTAGAAAGCAGTTCCGC‐3′ and reverse, 5′‐TGGGTAGGAGATGGAGATGC‐3′ primers. β‐actin was amplified using forward, 5′‐TCCCCCAACTTGAGATGTATGAAG‐3′, and reverse, 5′‐AACTGGTCTCAAGTCAGTGTACAGG‐3′ primers.

### 2.10 Transfection with siRNA

To investigate RNAi‐induced HIF‐1α knockdown in HK‐2 cells, 5.0 × 10^4^ HK‐2 cells per well were prepared in six‐well plates. Two kinds of RNAi—HIF‐1α siRNA (siHIF‐1α#1 [HSS104774, Thermo Fisher Scientific] and siHIF‐1α#2 [HSS104775, ThermoFisher Scientific])—were used with Lipofectamine RNAiMAX Transfection Reagent (Thermo Fisher Scientific). As a negative control, Stealth RNAi™ siRNA Negative Control Med GC Duplex #3 (12935‐113) was used; 1.5 µl of each siRNA and 5 µl RNAiMAX were mixed. siRNA‐transfected cells were incubated under normoxic or hypoxic (1% O_2_) conditions for 48 h, and RNA was extracted. The efficiency of HIF‐1α knockdown was examined using qRT‐PCR.

### 2.11 Live imaging of effects of a pH gradient

We visualized the intracellular pH gradient of living cells under a coverslip. Cells were treated with pHrodo Green AM Intracellular pH Indicator (p35373, ThermoFisher Scientific) according to the manufacturer's instructions, prior to implementing the coverslip model. An inverted fluorescence microscope, BZ‐X710 (Keyence Corporation) with a GFP filter was used to observe the time transition of the fluorescence intensity gradient under a coverslip.

### 2.12 Quantitative analysis of the HIF ring

#### 2.12.1 Site of the HIF ring

We measured the distance of the HIF rings and pimonidazole‐positive areas from the coverslip edges using ImageJ, as follows. The areas of the outer and inner circles of the HIF ring and pimonidazole‐positive circle were determined, and the radius of each circle was calculated. Each value was subtracted from 7.5 mm, which is the radius of a 15 mm coverslip, to calculate its distance from a coverslip edge. Three independent experiments of ICC of HIF in each condition were performed.

#### 2.12.2 Definition of the HIF ring

We used fluorescent images obtained from a coverslip model incubated under normoxia and neutral pH. We defined the site of the HIF ring and its outside and inside as 2.5–3.0 scale (0.22–0.45 mm), 0.5–1.0 scale (1.12–1.35 mm), and 5.0–5.5 scale (2.24–2.47 mm) from a coverslip edge, respectively, using ImageJ. We measured five sites of HIF‐1α signals per sample and calculated the average. Three independent experiments of ICC of HIF were performed.

### 2.13 Measurement of oxygen pressure by PLIM image

#### 2.13.1 Construction of a calibration line to measure oxygen pressure

A calibration curve of HK‐2 cells was made, based on the method described in our previous report (Yoshihara et al., 2015). HK‐2 cells loaded with 500 nM BTPDM1 for 30 min were cultured in an oxygen‐concentration‐changeable multi‐gas incubator equipped with an inverted fluorescent microscope that was connected to the lifetime measurement system. A calibration line based on Stern‐Volmer analyses was constructed using the phosphorescence lifetime (PL) of HK‐2 cells under several different oxygen tensions using Equation (1).

(1) $$\frac{1}{\tau_{p}}=\frac{1}{\tau_{0}}+k_{q}∙pO_{2}$$

where *τ_p_* represents the PL in *pO*_2_, *τ*_0_ represents the PL in deoxygenation, *k_q_* represents the quenching rate constant, and *pO*_2_ represents the partial pressure of oxygen. Using this calibration line, the oxygen pressure could be calculated from the PL.

#### 2.13.2 PLIM image of a coverslip model

HK‐2 cells loaded with 500 nM BTPDM1 for 30 min were prepared. A coverslip model was constructed and cultured in an O_2_ concentration‐changeable multi‐gas incubator equipped with an inverted fluorescent microscope, connected to the lifetime measurement system.

Phosphorescence lifetime imaging microscopy (PLIM) images were recorded using an inverted fluorescence microscope equipped with a confocal scanning system (Hirakawa et al., 2018). PLIM images were obtained after 30 min incubation in 21% O_2_ or 4% O_2_. Four PLIM images per sample, from the upper, bottom, left, and right regions near the coverslip edge, were taken, to determine the average PL, taking into account the variation in PLs in a coverslip.

#### 2.13.3 Identification of the HIF ring in a PLIM image

Pimonidazole was positive below 10 mmHg of oxygen pressure. The PL equivalent of 10 mmHg of oxygen pressure was 4034.6 ns, according to the calibration line, so the outer line of the pimonidazole circle in the PLIM image could be identified. Subsequently, the sites of the outer and inner HIF rings in the PLIM image could be found by using distance quantitative analysis. We measured the range of average PLs equivalent to the HIF ring in 21% O_2_ and 4% O_2_.

#### 2.13.4 Measurement of the oxygen pressure of the HIF ring

We calculated the range of oxygen pressures of the HIF ring from the PLs, using the calibration line. PLIM images were analyzed using SPCImage 5.0 (Becker & Hickl GmbH).

### 2.14 Statistical analysis

Dunnett's test was used to compare experimental and control groups, and *p*‐values < .05 were considered to indicate statistically significant differences. Student's *t*‐tests were used to compare three or more groups, and the Bonferroni adjusted *p*‐value was applied. All statistical analyses were performed using JMP Pro ver13.2.1 (SAS). Values were assumed to be derived from a normally distributed population, and are shown as mean ± standard deviation (SD).

## 3 RESULTS

### 3.1 Oxygen gradient formation in the hypoperfusion model

**FIGURE 1:**
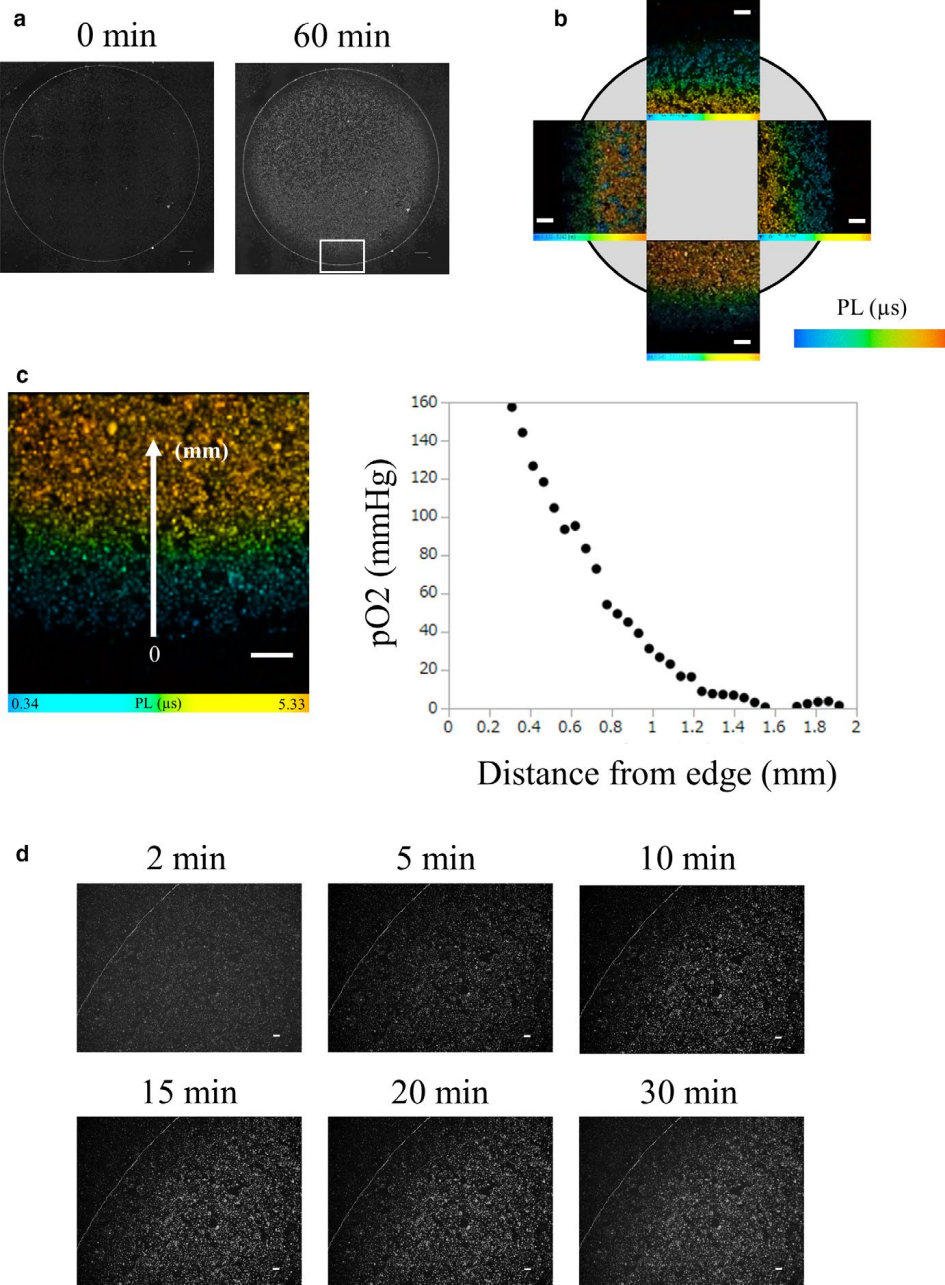
Oxygen gradient formation in the hypoperfusion model by coverslip placement. (a) Phosphorescence intensity of BTPDM1, which increased as the intracellular oxygen pressure of HK‐2 cells decreased, just after coverslip placement (left) and 60 min later (right). Phosphorescence intensity of BTPDM1 in HK‐2 cells 60 min after placement of a coverslip showed hypoxia in most of the inside and not at the edge of the coverslip (square). Scale bar: 1000 µm. (b) Phosphorescence lifetime imaging microscopy (PLIM) images of HK‐2 cells treated with BTPDM1 30 min after placement of a coverslip. Gray circle: a 15 mm round‐shaped coverslip, PL: phosphorescence lifetime, PL range: 0.32–5.24 µs (left image), 0.34–5.33 µs (bottom image), 0.08–5.11 µs (right image), and 0.12–5.06 µs (upper image). Scale bar: 500 µm. (c) Measurement of oxygen tension of HK‐2 cells around the edge of a coverslip PLs in PLIM images. The oxygen tension was calculated from a calibration line of HK‐2 cells (Figure S3). We analyzed the relationship between the oxygen tension and the distance from a coverslip edge. The oxygen tension decreased as the distance increased. The coverslip edge was determined to be zero. PL range: 0.34–5.33 µs, Scale bar: 500 µm. (d) Temporal profile of the phosphorescence intensity of BTPDM1 from HK‐2 cells covered with a coverslip. The oxygen gradient started forming 10 min after coverslip placement. Scale bar: 100 µm

We confirmed the existence of an oxygen gradient in the hypoperfusion model induced by coverslip placement by directly assessing oxygen tension, using phosphorescence. Phosphorescence intensity measurement can predict an approximate trend in oxygen pressure (Hirakawa et al., 2015; Yoshihara et al., 2015). We observed the phosphorescence intensity of a coverslip model of HK‐2 cells treated with BTPDM1. Images of the phosphorescence intensity showed hypoxia in most of the area inside the coverslip, but no hypoxia near the edge, an observation which suggested the existence of an oxygen gradient around the edge of the coverslip in HK‐2 cells covered with a coverslip (Figure 1a). Since the intensity of phosphorescence depends on the concentration of the probe, and on the excitation time, a phosphorescence lifetime (PL) measurement is useful for quantitative analysis of oxygen pressure (Yoshihara et al., 2015). Thus, for the quantitative measurement of the oxygen tension of cultured cells around the edge of a coverslip, we obtained PLIM images of HK‐2 cells covered with a coverslip for 30 min (Figure 1b). PL extended as the distance from the coverslip edge increased. We confirmed that the oxygen tension, calculated using a calibration line (Figure S3), decreased as the distance from the coverslip edge increased, providing evidence for the existence of an oxygen gradient around the edge of the coverslip (Figure 1c). Examination of the temporal profile of the phosphorescence intensity indicated that the oxygen gradient started to form 10 min after coverslip placement (Figure 1d).

### 3.2 Unique HIF‐1α distribution in the hypoperfusion model, “HIF ring”

**FIGURE 2:**
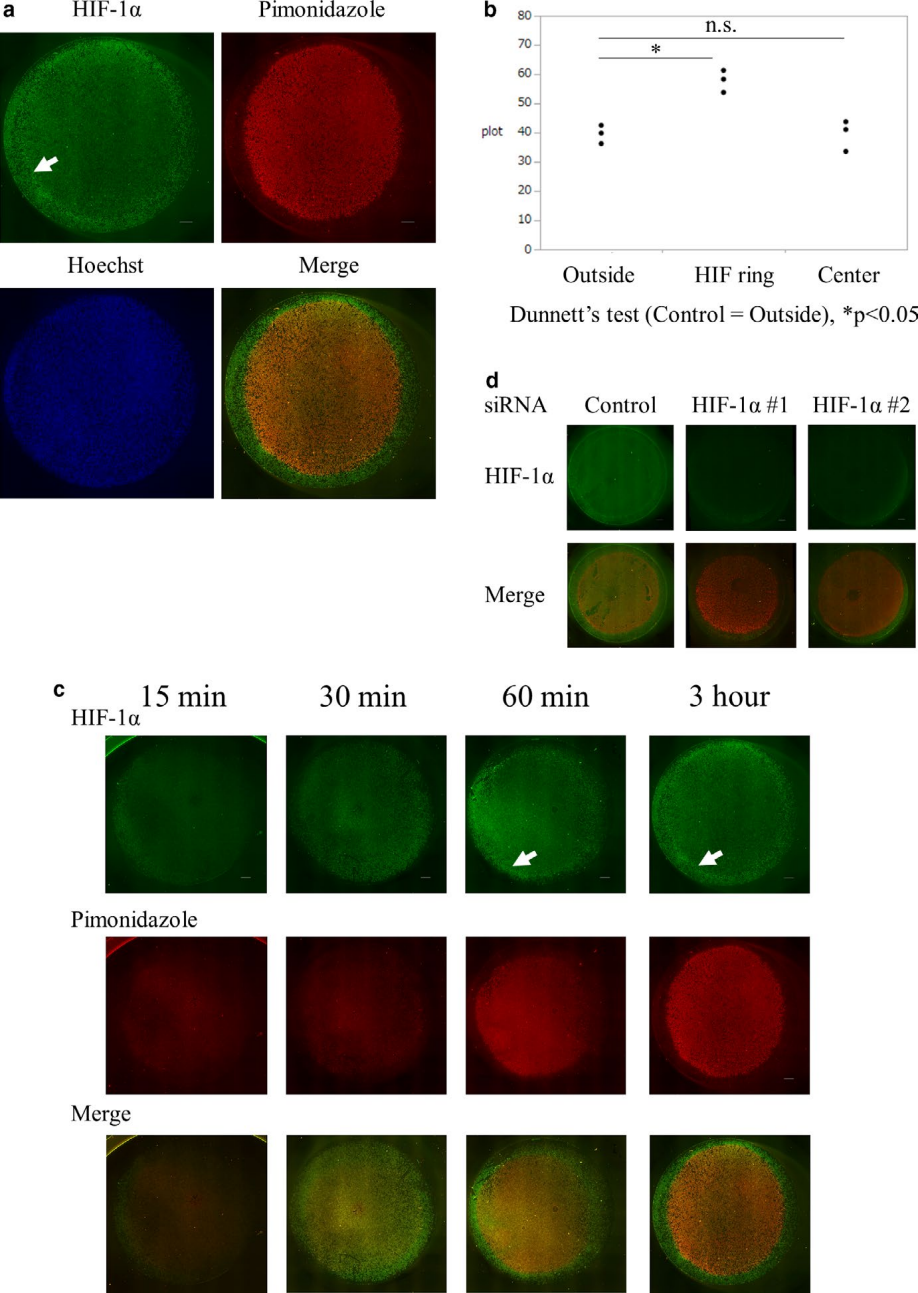
Unique HIF‐1α distribution in the hypoperfusion model, “HIF ring”. (a) Immunocytochemistry of HIF‐1α of HK‐2 cells covered with a round 15 mm coverslip for 3 h, counterstained with pimonidazole. HIF‐1α showed a doughnut‐shaped formation on the edge of the pimonidazole‐positive area (arrow). Scale bar: 1000 µm. (b) Quantitative analysis of HIF‐1α signals on the site of the HIF ring as well as its outside and inside. The intensity of the HIF‐1α signal was significantly higher on the HIF ring than on the outside or inside. (c) Temporal profile of ICC of HIF. The HIF ring (arrow) and pimonidazole‐positive area started to form 1 h after coverslip placement, and the HIF ring (arrow) appeared static in 3 h. Scale bar: 1000 µm. (d) ICC of HIF in HIF‐1α knockdown conditions. HIF‐1α signals were attenuated in the whole area of a coverslip under HIF‐1α knockdown conditions. Scale bar: 1000 µm

We examined the HIF‐1α distribution in the hypoperfusion model with an oxygen gradient. The ICC of HIF‐1α in HK‐2 cells showed a doughnut‐shaped enhancement on the edge of the pimonidazole‐positive area, which we called the “HIF ring” (Figure 2a). The intensity of the HIF‐1α signal was significantly higher on the HIF ring than in its inner or outer areas (Figure 2b). This phenomenon was confirmed using ICC with another HIF‐1α antibody (Figure S4a) or other tubular cell lines (Figure S4b,c). We also performed ICC analysis of HIF‐1α in other kinds of cultured cells, such as HeLa cervical cancer cells. Although the weak adhesion of these cells to a coverslip made detailed evaluation of HIF‐1α distribution difficult, we observed a doughnut‐shaped enhancement of HIF‐1α in HeLa cells (Figure S4d).

The HIF ring and pimonidazole‐positive area started forming several hours after coverslip placement, and the HIF ring appeared static by 3 h (Figure 2c). HIF‐1α knockdown resulted in the disappearance of the signal HIF‐1α, including the HIF ring (Figure 2d, Figure S4e), indicating that the HIF ring was dependent on HIF‐1α.

**FIGURE 3:**
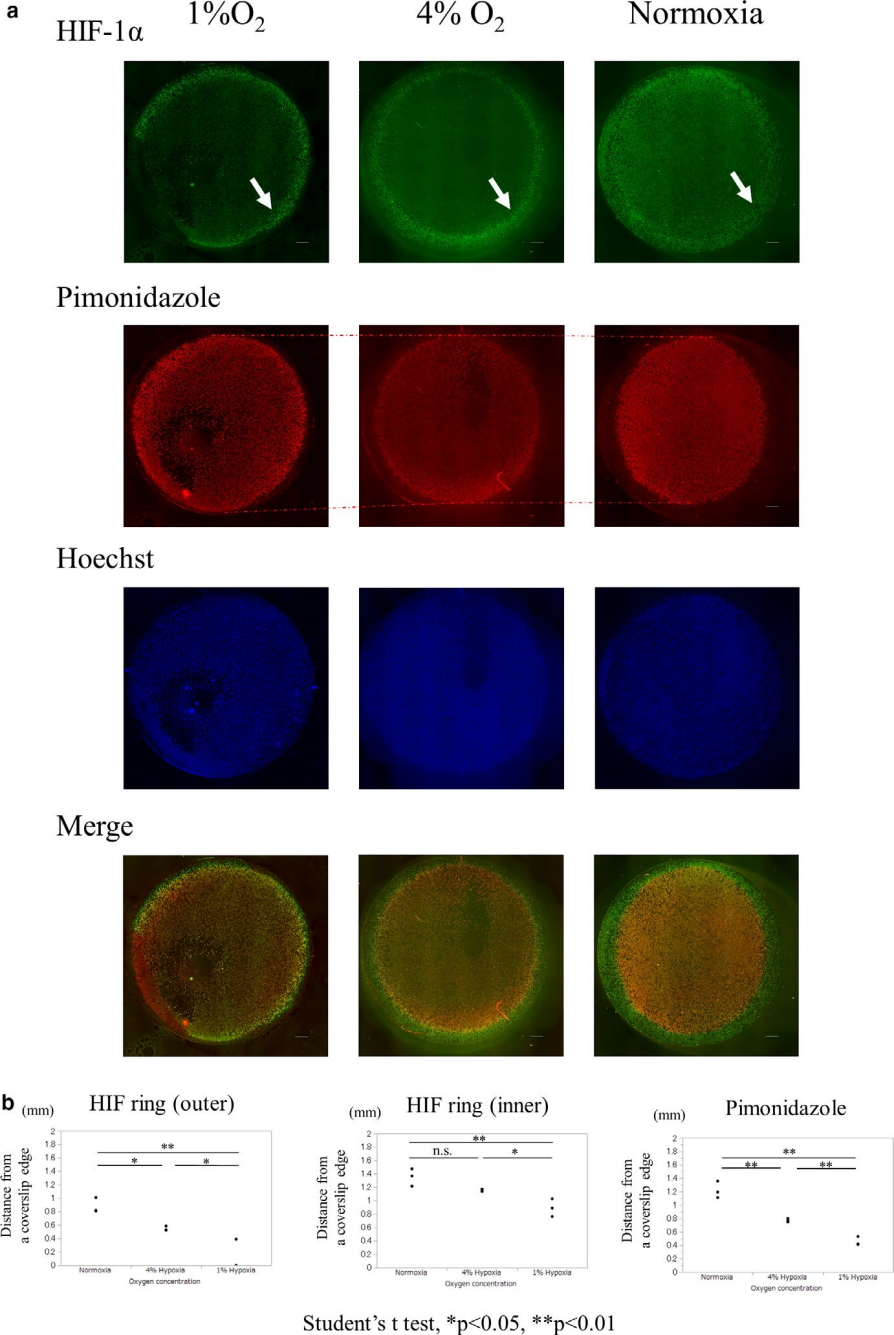
HIF‐1α distribution under different homogenous oxygen tensions incubation. (a) ICC of HIF of HK‐2 cells covered with a round 15 mm coverslip under different homogenous oxygen tensions, incubated for 3 h. Hypoxic incubation of a coverslip model induced the expansion of the HIF ring (arrow) and pimonidazole‐positive area outward (dotted line). Scale bar: 1000 µm. (b) Quantitative analysis of each distance of the HIF ring and pimonidazole‐positive area from a coverslip edge in normoxia, 4% O_2_, and 1% O_2_. Both the HIF ring and the pimonidazole‐positive area increased as oxygen tension decreased. The distance of the outer circle of the HIF ring was 0.88 ± 0.11 mm, and the pimonidazole‐positive area was 1.22 ± 0.13 mm, and that of the inner circle of the HIF ring from a coverslip edge was 1.35 ± 0.13 mm in normoxia. Distances were 0.55 ± 0.35 mm, 0.77 ± 0.03 mm, and 1.16 ± 0.02 mm in 4% O_2_ respectively. In 1% O_2,_ the distances were 0.13 ± 0.22 mm, 0.46 ± 0.07 mm, and 0.89 ± 0.13 mm respectively. Each value is shown as the mean ± SD. Scale bar: 1000 µm

To investigate the possibility that HIF ring formation is dependent on oxygen, we investigated the distribution of HIF‐1α in the coverslip model under incubation with different oxygen tensions. We placed a dish into hypoxic chambers with different oxygen tensions immediately after making a coverslip model. During hypoxic incubation, we observed that both the HIF ring and the pimonidazole‐positive area appeared to expand outward (Figure 3a). We measured the distance of each HIF ring and pimonidazole‐positive area from a coverslip edge under normoxia, 4% O_2_ and 1% O_2_ incubation. Both the HIF ring and the pimonidazole‐positive area expanded outwards as the surrounding oxygen tension decreased (Figure 3b). These results indicated that the HIF ring was dependent on HIF‐1α and oxygen tension.

In order to confirm that doughnut‐shaped accumulation is a specific phenomenon of HIF, we performed ICC with housekeeping genes in the coverslip model. The GAPDH and actin signals were maintained over the whole area of a coverslip, and were comparable between the region of the HIF ring and the other regions (Figure S5a,b).

### 3.3 Measurement of the range of oxygen pressure of the HIF ring

**FIGURE 4:**
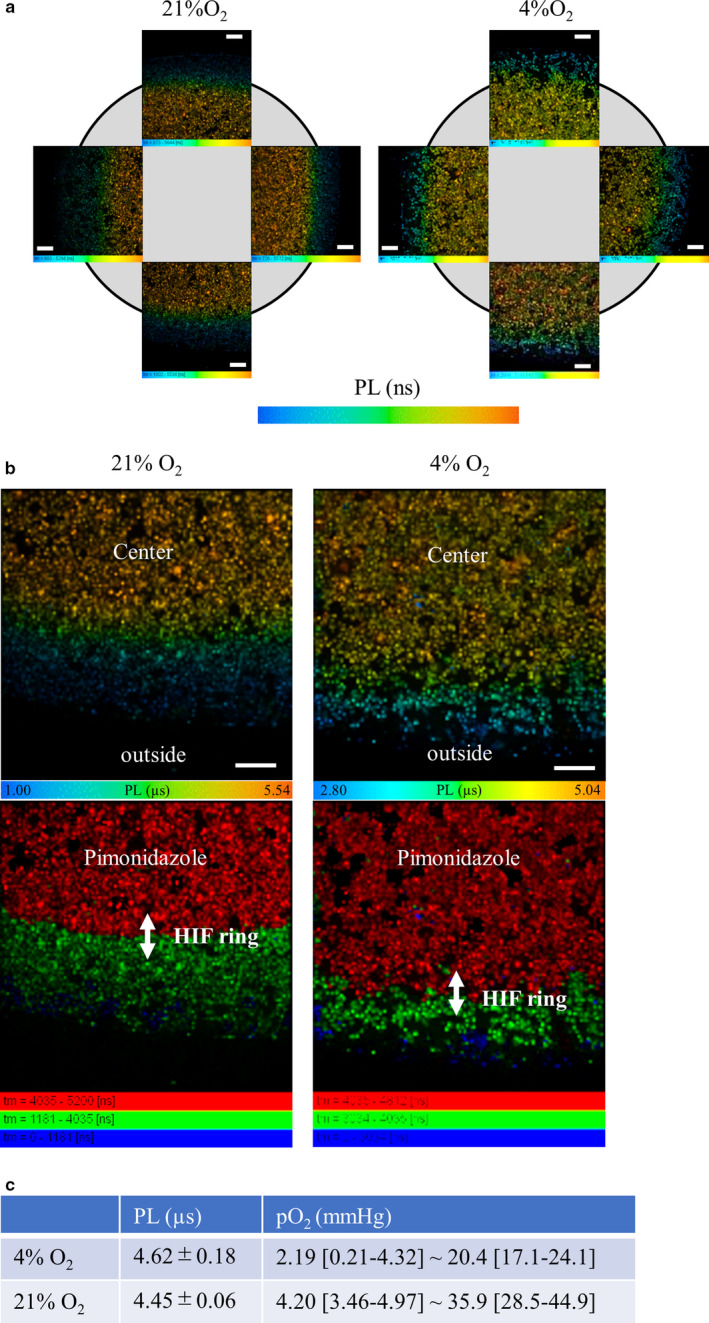
Measurement of the range of oxygen pressure of the HIF ring. (a) Phosphorescence lifetime imaging microscopy (PLIM) images of a coverslip model obtained after 30 min incubation in 21% O_2_ or 4% O_2_. Four PLIM images per sample, at the upper, bottom, left, and right regions near a coverslip edge, were taken to measure phosphorescence lifetimes (PLs). PL range: 0.89–5.30 µs (21%, left image), 1.00–5.54 µs (21%, bottom image), 0.73–5.57 µs (21%, right image), 0.87–5.64 µs (21%, upper image), 2.53–5.15 µs (4%, left image), 2.80–5.04 µs (4%, bottom image), 2.59–5.37 µs (4%, right image), and 2.42–5.65 µs (4%, upper image) Scale bar: 500 µm. (b) Identification of the HIF ring in the PLIM image Pimonidazole was observed to be positive below 10 mmHg of oxygen pressure. Based on a calibration line, the PL equivalent to 10 mmHg of oxygen pressure was 4034.6 ns (Figure S3, Table S2); therefore, the pimonidazole‐positive circle in the PLIM image could be identified (red region), and subsequently, the site of the outer and inner HIF ring in the PLIM image could be found by using our distance quantitative analysis (Figure 3b). PL range: >4.04 µs (21% and 4%, red), 1.18–4.04 µs (21%, green), <1.18 µs (21%, blue), 3.03–4.04 µs (4%, green), and <3.03 µs (4%, blue) Scale bar: 500 µm. (c) We measured the range of PLs of the HIF ring and then calculated the oxygen pressure in 21% O_2_ and 4% O_2_ using a calibration line (Figure S3) as 4.20 (3.46–4.97) ~35.92 (28.48–44.88) mmHg and 2.19 (0.21–4.32) ~20.44 (17.10–24.12) mmHg respectively. Each value is shown as mean and the subsequent parentheses show the mean ±SD range. pO_2_ represents the partial pressure of oxygen

We confirmed the ring‐shaped enhancement of HIF‐1α accumulation in cultured cells covered with a coverslip, a phenomenon dependent on oxygen tension. To determine the range of oxygen tension involved, we analyzed the oxygen pressure of the HIF ring using a phosphorescence lifetime technique. We obtained PLIM images of the coverslip model after 30 min incubation in 21% O_2_ or 4% O_2_ (Figure 4a). The HIF ring formed on the edge of the pimonidazole circle in the ICC of HIF (Figure 3b). We identified the site in the PLIM image that was equivalent to the pimonidazole‐positive area in the ICC of HIF, and subsequently identified the site corresponding to the HIF ring (Figure 4b), using quantitative analysis of the distance data from the HIF ring and the pimonidazole‐positive area (Figure 3b). We also measured the range of PLs of the HIF ring, and then used a calibration line (Figure S3) to calculate the oxygen pressure of the HIF ring in 21% O_2_ (4.20 [3.46–4.97] ~35.9 [28.5–44.9] mmHg), and 4% O_2_ (2.19 [0.21–4.32] ~20.4 [17.1–24.1] mmHg) (Figure 4c).

### 3.4 HIF‐1α accumulation under homogenous oxygen tension

**FIGURE 5:**
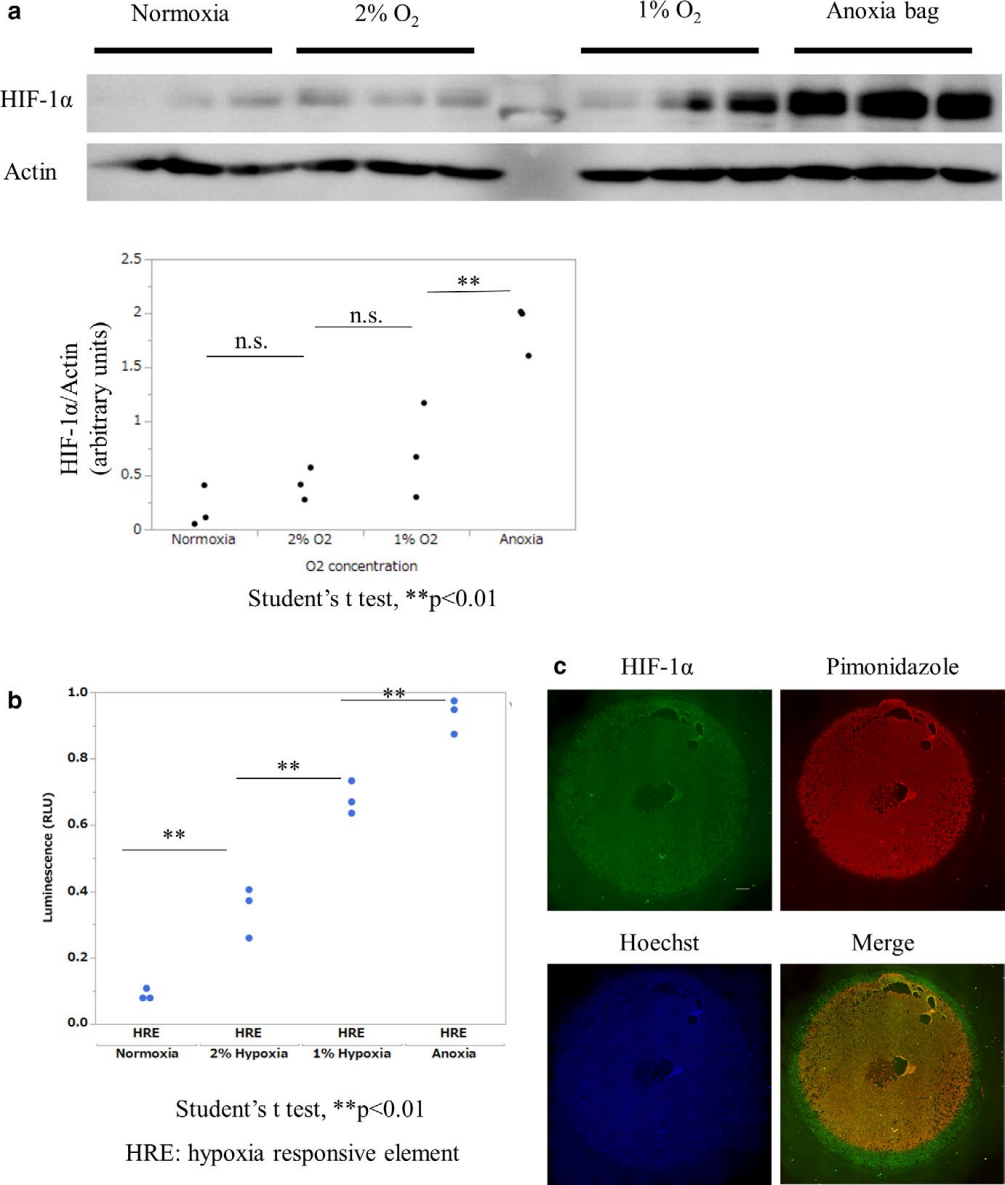
HIF‐1α accumulation in homogenous oxygen tension. (a) Quantitative analysis of HIF‐1α protein in different homogenous oxygen tensions by western blotting. Quantitative analysis of HIF‐1α bands normalized to those of actin showed that HIF‐1α accumulation significantly increased in anoxic incubation compared with normoxic incubation. (b) Quantitative analysis of HRE promoter activity in different homogenous oxygen tensions using an HRE‐luciferase reporter assay. HRE promoter activity increased as oxygen tension decreased. These results represent three independent experiments in each condition. (c) ICC of HIF of HK‐2 cells treated with cobalt chloride, a chemical inducer of HIF‐α. HIF‐1α signal did not increase inside the HIF ring, while the HIF ring became unclear because of the increased signal of HIF‐1α outside the HIF ring. Scale bar: 1000 µm

While weak signals of HIF‐1α outside the HIF ring in a coverslip model can be attributed to insufficient hypoxia, those inside the ring, where HIF‐1α should have been more strongly activated by the lower oxygen tension, must be controlled by an oxygen‐independent phenomenon. To examine the phenomena that cause a lack of maximum accumulation of HIF‐1α in the anoxic area occurring only in the hypoperfusion model, we examined whether there was an inverse relationship between oxygen tension and HIF‐1α accumulation under incubation with homogeneous oxygen tension. Western blot analysis of cell lysates under different homogeneous oxygen tensions showed an increase in HIF‐1α accumulation during anoxic incubation (Figure 5a). The HRE‐luciferase reporter assay also indicated that HIF‐1α accumulation was increased with lower oxygen tension under conditions of homogeneous oxygen tension (Figure 5b). These results indicated that accumulation of HIF‐1α was oxygen tension‐dependent, with the maximum accumulation observed in the anoxic range. The characteristics of HIF‐1α should vary between a hypoperfusion model and a model with homogenous oxygen tension. We speculated that the unique phenomenon of HIF‐1α accumulation in this hypoperfusion model might be determined by a factor other than oxygen tension.

### 3.5 HIF ring formation was independent of PHD

**FIGURE 6:**
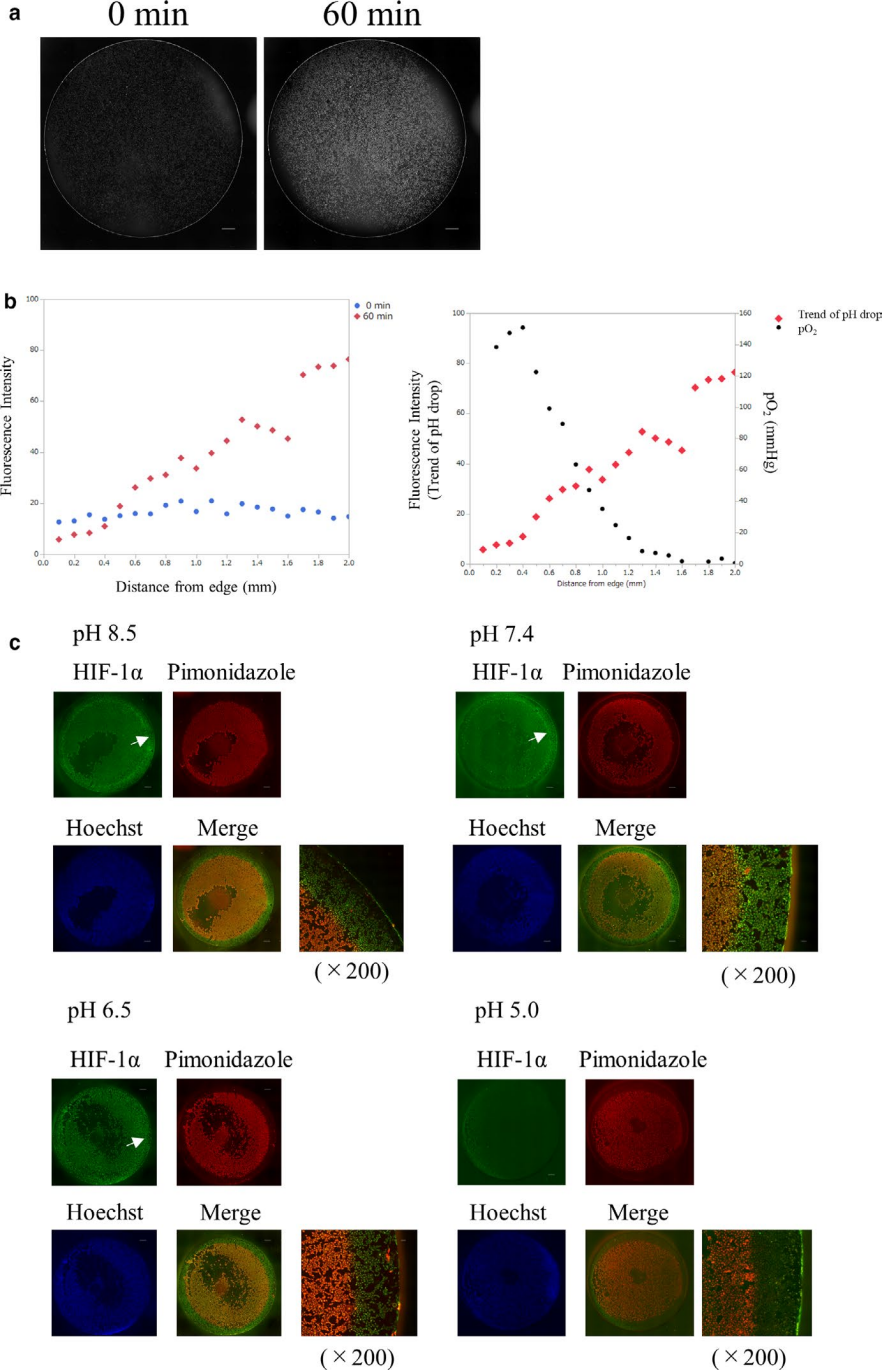
Impact of pH on HIF‐1α accumulation. (a) Live imaging of pH in coverslip model. HK‐2 cells were treated with pHrodo Green AM Intracellular pH Indicator, which showed strong fluorescence intensity as the intracellular pH dropped. Fluorescence intensity image of HK‐2 cells just after they were covered with a coverslip (left) and after 60 min (right) demonstrated that pH decreased in most of the inside area, but not near the edge of the coverslip. Scale bar = 1000 µm. (b) Correlation between fluorescence intensity of HK‐2 cells treated with pH indicator 60 min after placement of a coverslip, and the distance from a coverslip edge (left). The fluorescence intensity increased as the distance increased, which suggested a pH gradient around the coverslip edge. The decrease in oxygen tension (Figure 1c) and the trend of pH drop progressed as the distance from the coverslip edge increased, which demonstrated the existence of both oxygen and pH gradient around coverslip edges (right). (c) Comparison of ICC of HIF at pH 8.5, pH 7.4, pH 6.5, and pH 5.0. The HIF ring was clearly observed on the edge of the pimonidazole‐positive area at pH 8.5 or pH 7.4. The HIF ring existed but became obscure at pH 6.5 incubation (arrow). HIF‐1α signals were attenuated in the whole area of the coverslip model, and the HIF ring was not observed at pH 5.0. Scale bars: 1000 µm and 100 µm (×200)

We examined whether HIF‐1α degradation, a possible mechanism of HIF‐ring formation, was upregulated inside the HIF ring. This idea seemed to be invalid, because hydroxylation by PHD, a rate‐limiting process of HIF degradation, requires oxygen as a substrate. Cobalt chloride is widely used as a chemical HIF stabilizer. We constructed a coverslip model of HK‐2 cells treated with cobalt chloride, and performed ICC analysis of HIF. The HIF‐1α signal did not increase inside the HIF ring, which became unclear because of the increased signal of HIF‐1α outside the ring (Figure 6c). The ICC of HK‐2 cells with knockdown of PHD2, which is believed to be the primary HIF prolyl hydroxylase in cell culture experiments (Strowitzki et al., 2019), produced similar results (Figure S6).These results indicated that the PHD‐VHL axis of HIF degradation was unrelated to HIF ring formation.

### 3.6 Impact of pH on HIF‐1α accumulation

A previous report described the use of a coverslip model of cardiac myocytes that had a drop in pH inside the coverslip (Pitts & Toombs, 2004). We investigated the pH under a coverslip, and its impact on HIF ring formation. We treated HK‐2 cells with an intracellular pH indicator, the fluorescence intensity of which allows the evaluation of pH in the cells. Live imaging showed that the pH decreased over most of the inside area, but not near the edge of the coverslip, suggesting the existence of a pH gradient around the edge in the coverslip model (Figure 6a). Quantitative analysis of the correlation between fluorescence intensity and distance from a coverslip edge showed that the former was elevated as the distance increased, an observation which supported the existence of pH and oxygen gradients around the coverslip edge (Figure 6b). Western blot analysis of cell lysates under homogeneous oxygen concentrations found that incubation with pH 5.0 medium suppressed HIF‐1α accumulation under hypoxic conditions (Figure S7a–d). We also confirmed that HIF‐1α accumulation under hypoxia under different pH conditions decreased below pH 6.0 (Figure S8a–c).

**FIGURE 7:**
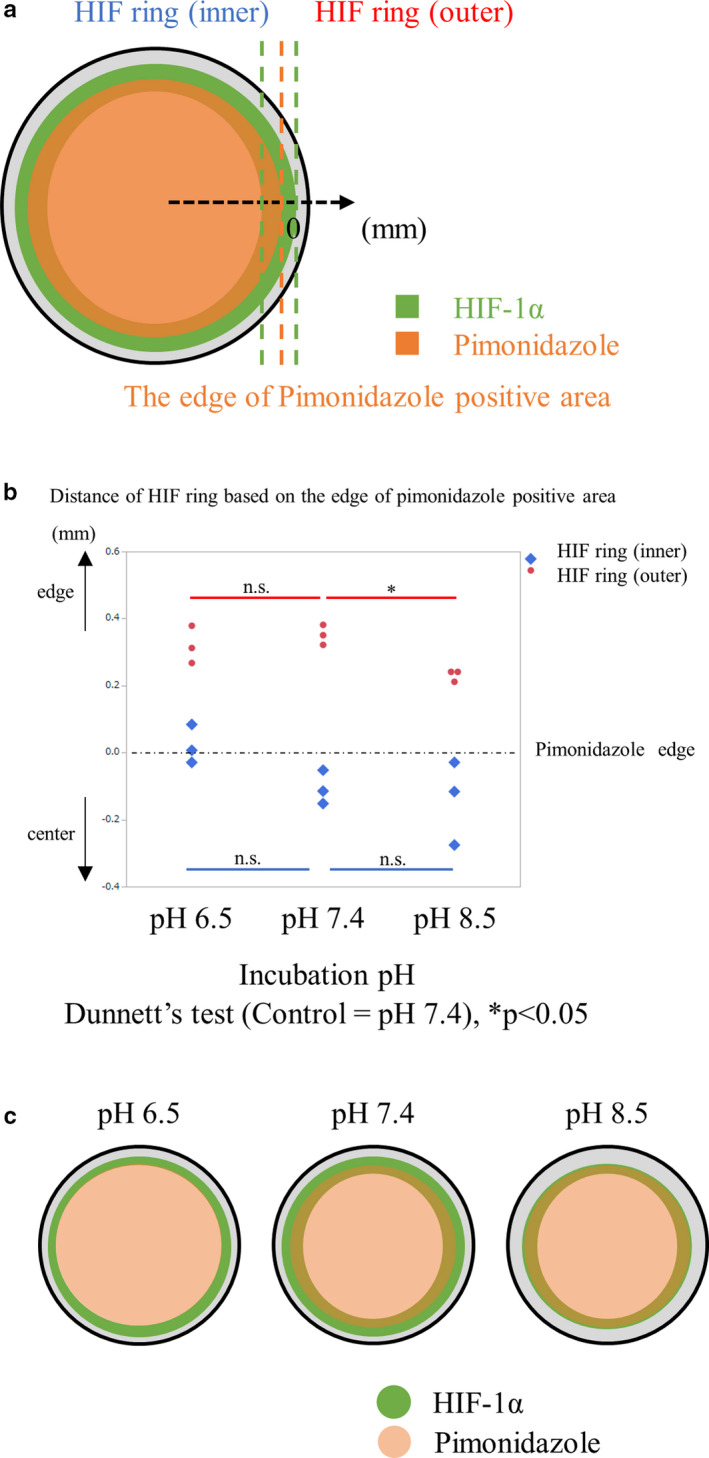
Quantitative analysis about the impact of pH on HIF ring. (a) Quantitative analysis of the variation in the positional relationship between the outer and inner edges of the HIF ring and the edge of the pimonidazole‐positive area in different pH incubations. We determined that the coordinate of the edge of the pimonidazole‐positive area was zero and the edge side of the coverslip was plus. (b) Compared with pH 7.4, the inner circle of the HIF ring expanded outwardly at pH 6.5, while the outer circle of the HIF ring shrank inwardly at pH 8.5 incubation, based on the edge of the pimonidazole‐positive area. At pH 5.0, the HIF‐1α signal was too weak for quantitative analysis of the HIF ring. (c) Diagram of ICC of HIF in different pH. The HIF ring moved outwards or inwards, depending on the acidity or alkalinity of the incubation. Gray circle: a 15 mm round‐shaped coverslip

One report emphasized the importance of mildly acidic conditions, at pH around 6.0. According to this study, reoxygenation after hypoxia acidified the media, which caused nucleolar sequestration of VHL. In turn, HIF degradation was prevented in C2C12 myotubes, PC12 neurons, and 786‐0 renal cancer cells (Mekhail et al., 2004). In our study, there was no change in VHL distribution in tubular cells between the regions with HIF‐1α accumulation and those in which it was suppressed, in a coverslip model with pH values from 5.0 to 7.4 (Figure S9a–c). Tubular cells are exposed to a wide range of pH conditions (Burke et al., 1999; Pavuluri et al., 2019; Raghunand et al., 2003), so we investigated the variation of the HIF ring in media of different pH values between 5.0 and 8.5. The HIF ring was clearly observed in media with pH 8.5 and 7.4 (Figure 6c). The HIF ring also existed at pH 6.5, but was obscured (Figure 6c). The HIF‐1α signal was attenuated in the whole area of a coverslip at pH 5.0. (Figure 6c). We performed further quantitative analysis of the positional relationship between the HIF ring and the edge of the pimonidazole‐positive area (Figure 7a), and discovered that the position of the ring varied depending on the pH. The inner circle tended to expand outward at pH 6.5, compared with pH 7.4, while the outer circle significantly shrank inward at pH 8.5, based on the edge of the pimonidazole‐positive area (Figure 7b,c). We also confirmed that the activities of housekeeping genes were maintained in a coverslip model under acidic incubation (Figure S10). Therefore, pH appears to play an important role in the mechanism underlying HIF ring formation.

## 4 DISCUSSION

**FIGURE 8:**
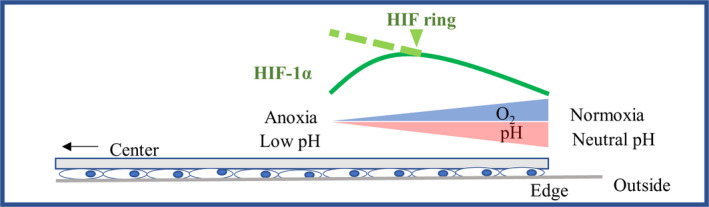
Possible mechanism of HIF ring formation. There is a pH gradient as well as an oxygen gradient at the coverslip edge in the hypoperfusion model, induced by coverslip placement. Although HIF‐1α accumulates as oxygen pressure decreases, HIF‐1α accumulation is suppressed because of low pH within a certain distance from the coverslip edge

In this study, we identified a unique distribution of HIF‐1α in renal tubular cells, using a model of hypoperfusion induced by coverslip placement. We found that the formation and maintenance of the HIF ring was regulated by oxygen pressure and pH, both of which existed in a gradient in the coverslip edge of a coverslip model. Based on these results, we propose a possible mechanism for HIF ring formation, involving HIF‐1α accumulating with decreasing oxygen tension, and the accumulation being suppressed because of low pH within a certain distance from a coverslip edge (Figure 8).

Hypoperfusion is induced by rarefaction of microvasculature in CKD (Mimura & Nangaku, 2010; Nangaku, 2006). In our previous work, we used *in vivo* imaging to demonstrate the presence of an oxygen gradient in renal tubular cells of normal mouse kidneys (Hirakawa et al., 2018). Oxygen tension is also expected to be heterogeneous in tubular cells in CKD. However, it remains unclear whether the existence of hypoperfusion with an oxygen gradient affects the defense system against hypoxia, HIF. In this study we investigated HIF distribution in cultured proximal tubule cells under a hypoperfusion model, and discovered that the oxygen gradient in cultured renal tubular cells was successfully modeled using a round glass coverslip. Evidence from experiments using homogeneous oxygen tension indicated that HIF‐1α, whose hydroxylation and subsequent degradation are mainly oxygen‐dependent, accumulated. That is, the amount of HIF‐1α increases as the oxygen tension decreases. In our coverslip model, however, HIF‐1α was suppressed in the anoxic area and had the highest accumulation at a certain distance from a coverslip edge. This doughnut‐shaped HIF accumulation has never been shown before. We showed that HIF ring formation could be observed regardless of the oxygen tension of the incubating atmosphere, but its location was dependent on the oxygen tension. The range of oxygen tension on the HIF ring was measured at approximately 4–20 mmHg using PLIM with BTPDM1. Most previous studies, which were focused on cultured cells in an atmosphere with a homogenous oxygen tension, found that the amount of HIF‐1α protein increased in the hypoxic or anoxic ranges (Ameri et al., 2002; Carrera et al., 2014). Thus, in our model, HIF ring formation must have been affected by a factor other than the oxygen tension; it was independent of PHD, a rate‐limiting process of HIF degradation. A breakthrough in elucidation of the mechanism of HIF ring formation was our discovery of the role of the pH gradient, as well as the oxygen gradient, in the coverslip model, which limited the inflow of the media similar to an *in vivo* hypoperfusion model. In this hypoperfusion model, the intracellular pH decreased as the distance from the coverslip edge increased, and a pH gradient was observed around the coverslip edge. We showed that HIF‐1α accumulation was suppressed at low pH, around pH 5.0, compared with incubation at around neutral pH under homogeneous oxygen tension. The outer edge of the HIF ring expanded outward at pH 6.5, and the inner edge of the HIF ring shrank inward at pH 8.5, based on the edge of the pimonidazole‐positive area. The HIF ring was obscured under acidic conditions, and the HIF‐1α signal vanished at pH 5.0. Thus, we elucidated the importance of pH for HIF ring formation in a coverslip model. We demonstrated the possibility that the HIF ring was formed by the suppression of HIF‐1α accumulation by low pH inside the ring.

Several previous studies using the coverslip method have used cancer cells. According to one report using the human hepatoma cell line Hep3B, which expresses the oxygen‐dependent red shift of green fluorescent protein (AcGFP1), live imaging of cells covered with a rectangular coverslip showed a red shift as the distance from a coverslip edge increased (Takahashi & Sato, 2010). The emission spectrum of hepatoma cells shifted from the wavelength of green fluorescent protein (GFP) fluorescence to that of red as the oxygen tension decreased. In another study, the oxygen tension of SCC‐7 cells with a round 15‐mm coverslip was measured using a phosphorescence lifetime technique. The oxygen tension was 6.9 mmHg and 166 mmHg, calculated from PL, which was 3.89 µs and 893 µs, 0–2 mm inside, and 0–1 mm outside of the coverslip edge, respectively (Yoshihara et al., 2015). The coverslip method has also been used with cardiac myocytes, a system which has attracted attention as a promising *in vivo* ischemia‐reperfusion model. Kelly et al showed that myocytes covered with a coverslip underwent marked morphological changes over time, accompanied by alterations in mitochondrial membrane potential and plasma membrane dynamics, eventually resulting in myocyte death. They also demonstrated that the intracellular pH of myocytes covered with a coverslip drops rapidly to approximately pH 4 in the center of a coverslip (Pitts & Toombs, 2004). A coverslip model, inhibiting oxygen or nutritional diffusion to cultured cells under a coverslip, can mimic an *in vivo* model of ischemic, normal, and marginal zones in the center, outside, and edge of a coverslip respectively. Several studies have used this model as an ischemia‐reperfusion model of myocytes *in vitro* (Chun et al., 2015; Pitts et al., 2008; Solhjoo & O'Rourke, 2015; Wang et al., 2012). To the best of our knowledge, our study is the first to apply a coverslip method to tubular cells. In our system, the distance from a coverslip edge to a region equivalent to 10 mmHg of oxygen tension, where pimonidazole should turn positive, was 1.22 mm, and oxygen tension decreased to as low as zero within a distance of 2 mm from the coverslip edge. This result may be compatible with previous reports using a round 15 mm coverslip, demonstrating the existence of an oxygen gradient at least within 2 mm from the edge (Akiyama et al., 2018; Yoshihara et al., 2015). We examined the rate of apoptosis of cells under coverslips (Figure S11) and discovered that GAPDH and actin were maintained even inside the HIF ring, indicating that the HIF ring was not caused only by cell apoptosis or death inside the HIF ring.

Several studies have measured the oxygen tension in the kidneys using different methods. A few examples of the oxygen tension measurement of normal kidneys were as follows: 50 mmHg and 30 mmHg in the cortex and the medulla using microelectrodes; and 49 mmHg and 41 mmHg in the S1 and S2 segments of tubular cells of the cortex using PLIM (Hirakawa et al., 2017, 2018; Zhang et al., 2014). The oxygen tension of sick kidneys would be lower. According to a previous report using oxygen microelectrodes, diabetic rats had lower renal parenchymal oxygen tension: 36 mmHg and 11 mmHg in the cortex and medulla respectively (Heyman et al., 2013; Palm et al., 2003). Since oxygen microelectrodes measure the average parenchymal oxygen tension, a wider range is expected in sick kidneys. The intracellular oxygen tension decreased to zero mmHg in a model of an ischemic kidney whose lateral renal artery and vein were clamped (Hirakawa et al., 2015). Considering these findings, the range of oxygen tension on the HIF ring, approximately 4–20 mmHg, seemed to be plausible *in vivo*.

We further discovered that pH affected HIF ring formation as well as oxygen tension. Since tubular epithelial cells in the kidney are continuously exposed to urinary fluid, the pH of tubular cells should be affected by that of urine. Considering the wide range of pH in urine, we decided to study cells incubated at a pH range of 5.0–8.5. There have been studies into the range of pH in normal kidneys. One study found that the pH of the cortex was 7.39 ± 0.08, and that of the medulla was 7.20 ± 0.09, as measured using microelectrodes (Burke et al., 1999). Another study, using MRI‐based pH imaging, showed pH values of 7.3 ± 0.13 and 7.0 ± 0.29 respectively (Raghunand et al., 2003). It was reported that renal pH decreased from 6.5 to 6.32, and to as low as 5.83 in a severe case, in a mouse model of CKD with acidosis (Pavuluri et al., 2019). These observations have provided evidence of pH drop and variation in CKD. However, the impact of pH on HIF‐1α induced by chronic hypoxia in CKD has not been sufficiently well‐examined. In the present study, a shift of the HIF ring was observed between pH 6.5 and pH 7.4, a pH range that is plausible in CKD. This result supported the hypothesis that a mild decrease in pH affects HIF‐1α accumulation in CKD. Based on the evidence of a pH drop below 6.0 in severe cases of CKD, it is also necessary to assess the HIF‐1α physiological state at lower pH. Although there have been reports that an acidic environment, even in normoxia, stabilizes HIF‐1α, most of these reports investigated conditions of mild acidity, over pH 6.0 (Filatova et al., 2016; Mekhail et al., 2004). In this work, we showed suppression of HIF‐1α accumulation in tubular cells at pH 5.0, and attenuation of HIF‐1α signals in a coverslip model incubated at pH 5.0. Therefore, renal pH decrease in CKD *in vivo* might be correlated with insufficient HIF activation, as well as with uremic toxins in the hypoxic kidney, aggravating CKD progression (Tanaka et al., 2013).

Our study has several limitations. First, in the area of hypoperfusion, the culture media should be deficient in nutrients, in addition to the presence of oxygen deficiency and decreased pH. However, it is difficult to delineate the effects of pH, oxygen tension, and other factors induced by hypoperfusion. In the body, ischemia, a pathological hypoperfusion of the blood caused by a combination of low oxygen and low nutrients, occurs in organs and cells. It is important to understand the differences between hypoxia sensing and ischemia sensing. Despite the difficulty in elucidating the biological effect of each factor, our hypoperfusion model is physiologically plausible, mimicking the pathological status *in vivo*. Second, making a coverslip model requires skill and practice, since the majority of cells occasionally became detached during removal of a coverslip, especially when using the traditional method (Figure S2a). This issue depended on the cell line used. For example, it was difficult for us to apply a coverslip model to HEK 293 cells, because their adhesion to a coverslip was relatively weak. Third, it was desirable to minimize damage to cultured cells under a coverslip. The number of damaged cells increased as the period during which they were covered increased, as shown by the analysis of apoptosis. We determined that 3 h was appropriate for estimating the HIF ring when it seemed to be static, and approximately as few as 10% of cells became apoptotic (Figure S11). The fourth limitation was the difficulty of attaching cultured cells to a coverslip in a uniform way in every sample (Figure S2b,c). The difference in attachment between a traditional and an alternative method of making a coverslip model could also have affected the study. We measured the range of oxygen tension equivalent to the HIF ring by a phosphorescence lifetime technique, in which we created a coverslip model using a traditional method. Since the HIF ring during immunocytochemistry was visualized using an alternative method, the true oxygen tension might be different. We minimized these errors by using the evidence that pimonidazole was positive at 10 mmHg or less of the oxygen tension. The fifth limitation is that intracellular pH is not necessarily the same as the pH of the culture medium. Several past reports have shown that intracellular pH is consistent with medium pH to a certain extent in cultured cells (Michl et al., 2019), we could assess only the trend in intracellular pH by changing the medium pH. *In vivo*, the relationship between the pH of intracellular and extracellular regions, such as urine or body fluid, is more complicated than in cultured cells. Thus, further studies about the way in which pH shift regulates the accumulation of HIF‐1α protein *in vivo* will be necessary in the future.

Another limitation is that pH should have an effect on the hypoxia marker, pimonidazole. We compared the distance of the edge of the pimonidazole‐positive area from the center of a coverslip under different pH conditions in our coverslip model. The edge of the pimonidazole‐positive area was comparable between pH 6.5, 7.4, and 8.5 (Figure S12a,b). A previous study also demonstrated the maintenance of pimonidazole binding at different pH values (Kleiter et al., 2006). Based on this evidence, we concluded that pimonidazole is an appropriate hypoxia marker for our coverslip experiments. The final limitation is that, given a range of oxygen tension between approximately 4 mmHg and 20 mmHg (0.52% O_2_ to 2.6% O_2_) of the HIF ring, Pt(II)‐ and Pd(II)‐porphyrins, which are widely used as biological oxygen probes, have advantages for measuring very low oxygen concentrations because of their significantly longer phosphorescence lifetimes compared with those using Ir(III) complexes (Yoshihara et al., 2017). However, in general, a quantitative oxygen measurement based on phosphorescence, calculated using the Stern–Volmer equation, has better performance at low O_2_ ranges (Papkovsky & Zhdanov, 2016). Several previous studies have demonstrated the Ir(III) complex‐based measurement of oxygen tensions as low as approximately 10 mmHg (Akiyama et al., 2018; Yoshihara et al., 2015). Therefore, we believe that the range of oxygen tension of the HIF ring should be a precise result.

## 5 CONCLUSIONS

In summary, we found that understanding the roles of both oxygen and pH is essential for understanding the HIF‐1α physiological state in CKD, and can be gained by investigating tubular cells with hypoperfusion. The coverslip model, with its limitations of the inflow of media, was a good model of hypoperfusion *in vivo*, especially in the tubules in CKD, since rarefaction of the peritubular capillaries is a major hallmark of CKD (Mimura & Nangaku, 2010; Nangaku, 2006). This hypoperfusion model can also mimic the oxygen and pH gradients *in vivo*, such as tumors and ischemic lesions. The model provides a promising approach to the elucidation of these biological mechanisms.

## CONFLICT OF INTEREST

All authors have no conflicts of interest to declare.

## AUTHORS' CONTRIBUTIONS

All authors contributed to the formation of the overall concept. T.H. performed the overall experiments. T.H. and Y.H. analyzed the results and made the figures. T.H. wrote the original manuscript. K.M., T.Y., and S.T. performed the experiment using a phosphorescence lifetime technique and edited the part of the manuscript. Y.H., T. T., and M.N. edited the overall manuscript. All the authors have read and approved the final manuscript.

## Supporting information



Supplementary Material
