# Supplementary material for: A distinctive distribution of hypoxia‐inducible factor‐1α in cultured renal tubular cells with hypoperfusion simulated by coverslip placement
Source: Physiol Rep. 2020 Dec 28;9(1):e14689. doi: 10.14814/phy2.14689 (PMC7769172; doi:10.14814/phy2.14689)

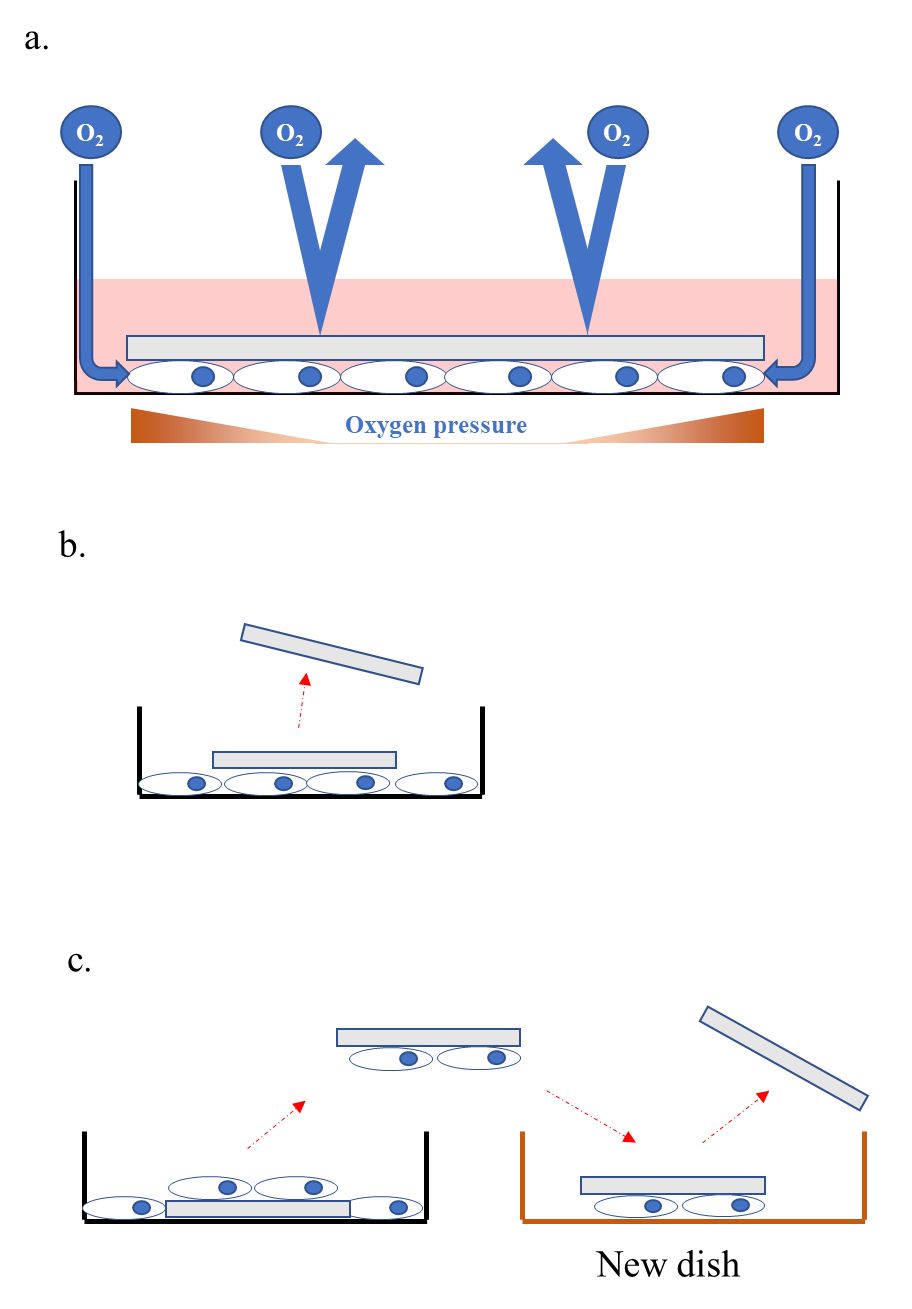


**Fig. S1: Constructing a hypoperfusion model by coverslip placement**

1. Diagram of a coverslip model. An oxygen gradient is formed due to the prevention of oxygen diffusion in monolayered cultured cells covered with a coverslip.
2. Traditional method of making a coverslip model. Cells are seeded on the bottom of a dish and a coverslip is placed on the cultured cells.
3. An alternative method for making a coverslip model. Cells are cultured on a coverslip, which is then inverted to attach the surface of cells on a coverslip to the bottom of a dish.


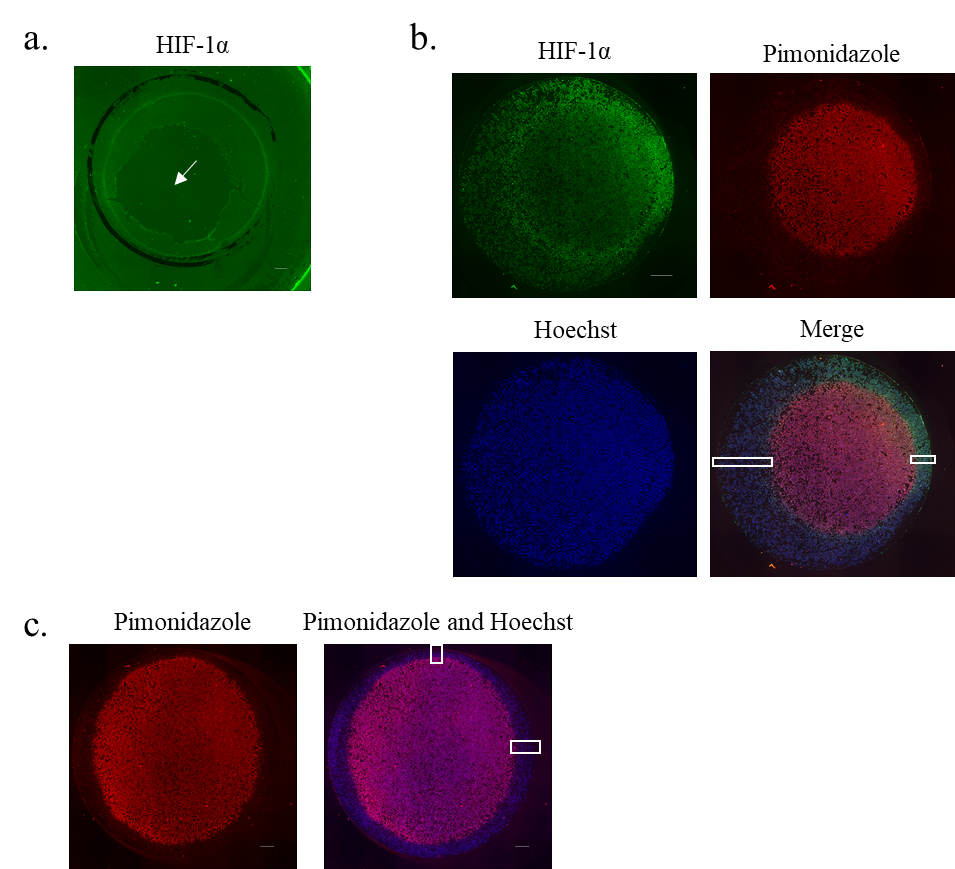


**Fig. S2: Limitations of a coverslip model**

1. Example of ICC of HIF using a traditional method. The majority of cells frequently detached from the bottom of a dish (arrow) upon coverslip removal for cell fixation in a traditional way. Scale bar: 1000 μm
2. ICC of HIF of a 10 mm round-shaped coverslip model. The attachment of HK-2 cells between the coverslip and the bottom of a dish was not uniform, as shown in the different sizes between the two squares. Scale bar: 1000 μm
3. ICC of pimonidazole-protein adduct in a 15 mm round coverslip model (Fig. 2a). The merged image of pimonidazole and Hoechst shows that the distance of the pimonidazole-positive area from a coverslip edge was not uniform (different sizes between two squares). Scale bar: 1000 μm


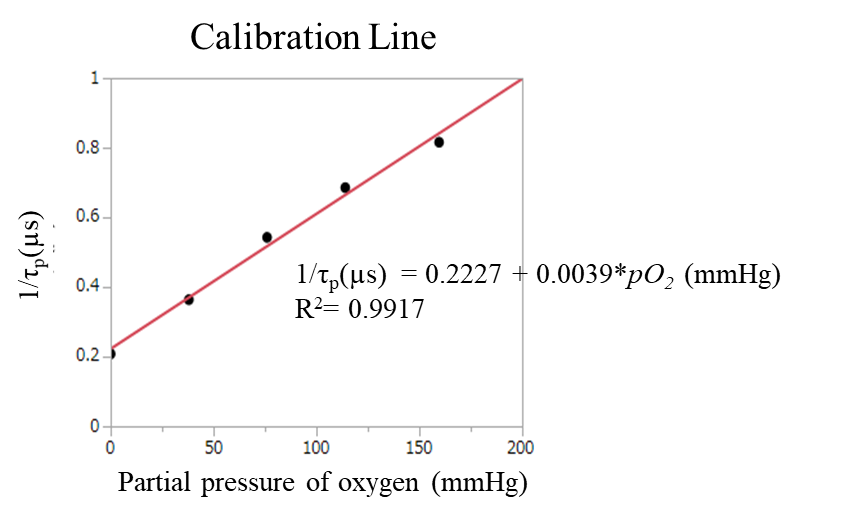


**Fig. S3: Calibration line of HK-2 cells treated with BTPDM1**

A calibration line based on Stern-Volmer analyses was plotted using the phosphorescence lifetime (PL) data of HK-2 cells loaded with BTPDM1 under several different oxygen tensions. *pO2*: partial pressure of oxygen. τ_p_: PL in *pO2*


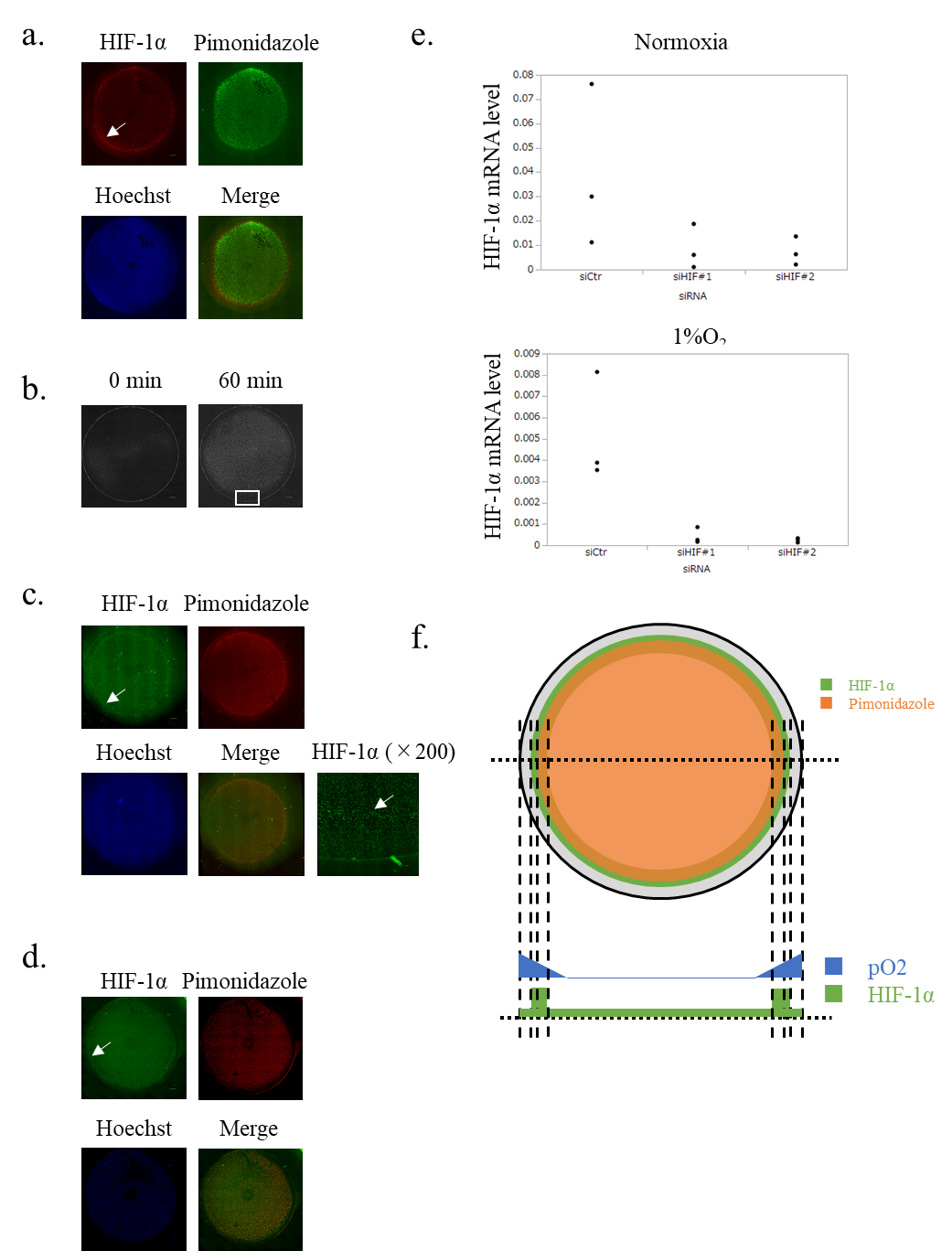


**Fig. S4: Supplementary experiments into ICC of HIF**

1. ICC of HIF of HK-2 cells using an anti-HIF-1α antibody. HIF-1α distribution showed a doughnut-shaped formation, which we named the HIF ring (arrow). Scale bar: 1000 μm
2. Phosphorescence intensity of BTPDM1 of renal proximal tubule epithelial cells (RPTEC) just after coverslip placement (left) and 60 min later (right). The phosphorescence intensity increased in most of the inside but not near the edge of a coverslip, indicating a hypoxic gradient around a coverslip edge (square) in RPTEC, 60 min after placement of a coverslip. Scale bar: 1000 μm
3. ICC of HIF of RPTEC. The HIF ring (arrow) was observed in the RPTEC as in HK-2 cells. Scale bars: 1000 μm and 100 μm (×200)
4. ICC of HIF of HeLa cervical cancer cells The HIF ring was observed outward in HeLa cells (arrow), compared with HK-2 cells (Fig. 2a). Scale bar: 1000 μm
5. qRT-PCR results of HIF-1α knockdown efficiency. HK-2 cells were transfected with two kinds of HIF-1α siRNA, siHIF-1α#1 and siHIF-1α#2. qRT-PCR results showed that knockdown efficiency was 78.0% and 81.2% under normoxia and 91.8% and 95.3% under 1% O_2_, respectively.
6. Diagram of ICC of HIF in a coverslip model. There was a pimonidazole-positive circle inside a round-shaped coverslip. HIF-1α showed a doughnut shape formation, which we named the HIF ring, on the edge of the pimonidazole-positive area.


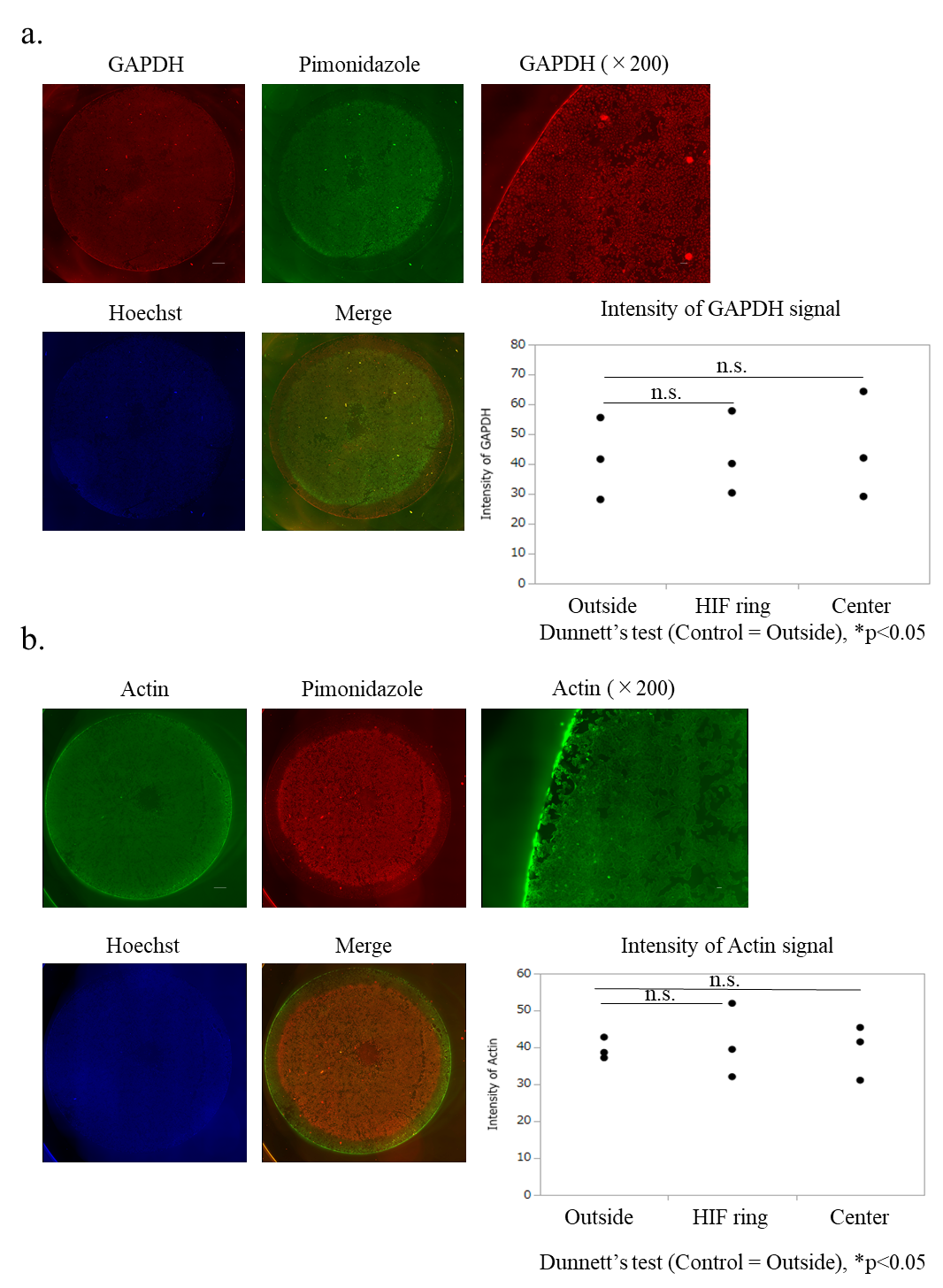


**Fig. S5: Immunocytochemistry with GAPDH in a coverslip model**

1. Immunocytochemistry with GAPDH of HK-2 cells covered with a 15 mm round coverslip for 3 h, counterstained with pimonidazole. GAPDH signals were maintained over the whole area of the coverslip (left). The intensity of GAPDH signals was comparable between the HIF ring and its outer or inner regions (right). Scale bars: 1000 μm and 100 µm (×200)
2. Immunocytochemistry of actin in HK-2 cells covered with a 15 mm round coverslip for 3 h, counterstained with pimonidazole. Actin signals were maintained in the entire area of the coverslip (left). The intensity of the actin signals was comparable between the HIF ring and its outer or inner regions (right). Scale bars: 1000 μm and 100 µm (×200)


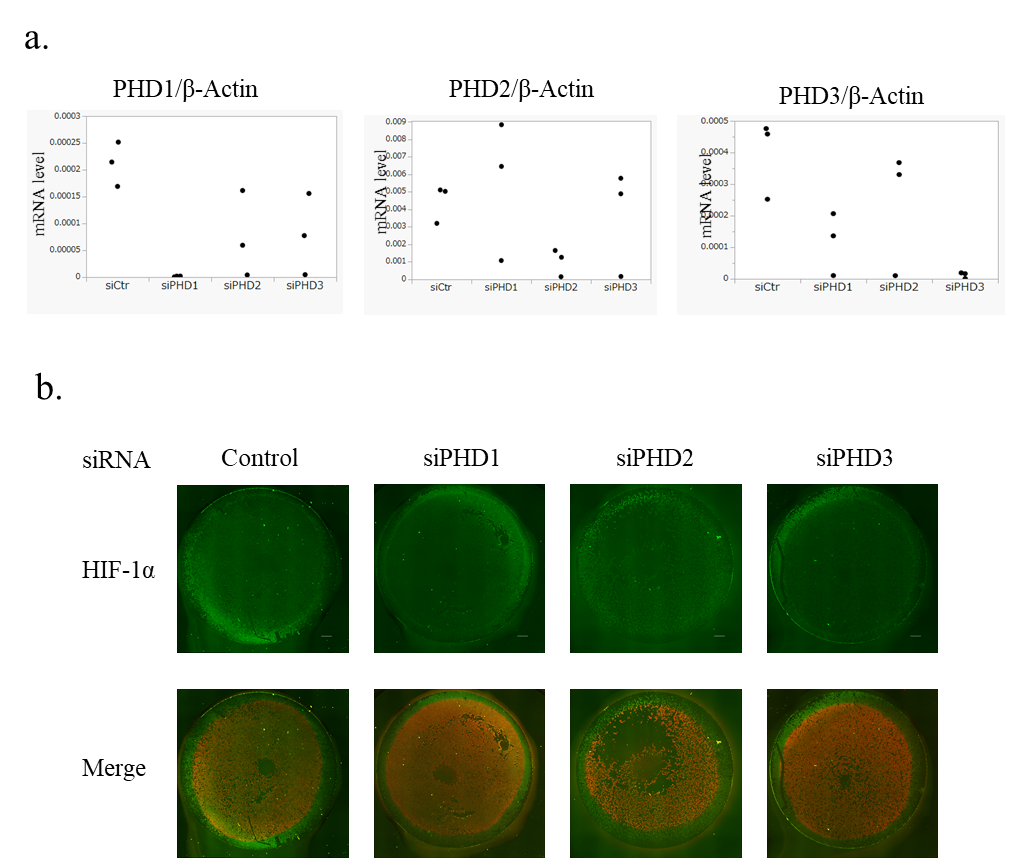


**Fig.S6: Immunocytochemistry in HK-2 cells with PHD knockdown conditions**

1. qRT-PCR results of the efficiency of prolyl hydroxylase domain (PHD) knockdown. HK-2 cells were transfected with siRNA against three subtypes of PHD, siPHD1, siPHD2, and siPHD3. qRT-PCR results showed that the knockdown efficiency of PHD1, PHD2, and PHD3 was 99.3%, 77.0%, and 96.9%, respectively.
2. ICC of HIF-1α in PHD1, PHD2, or PHD3 knockdown conditions. The HIF ring was observed in the PHD1 or PHD3 conditions. In the PHD2 knockdown condition, the HIF-1α signal did not increase inside the HIF ring, which became unclear because of the increased signal of HIF-1α outside the ring. Scale bar: 1000 μm.


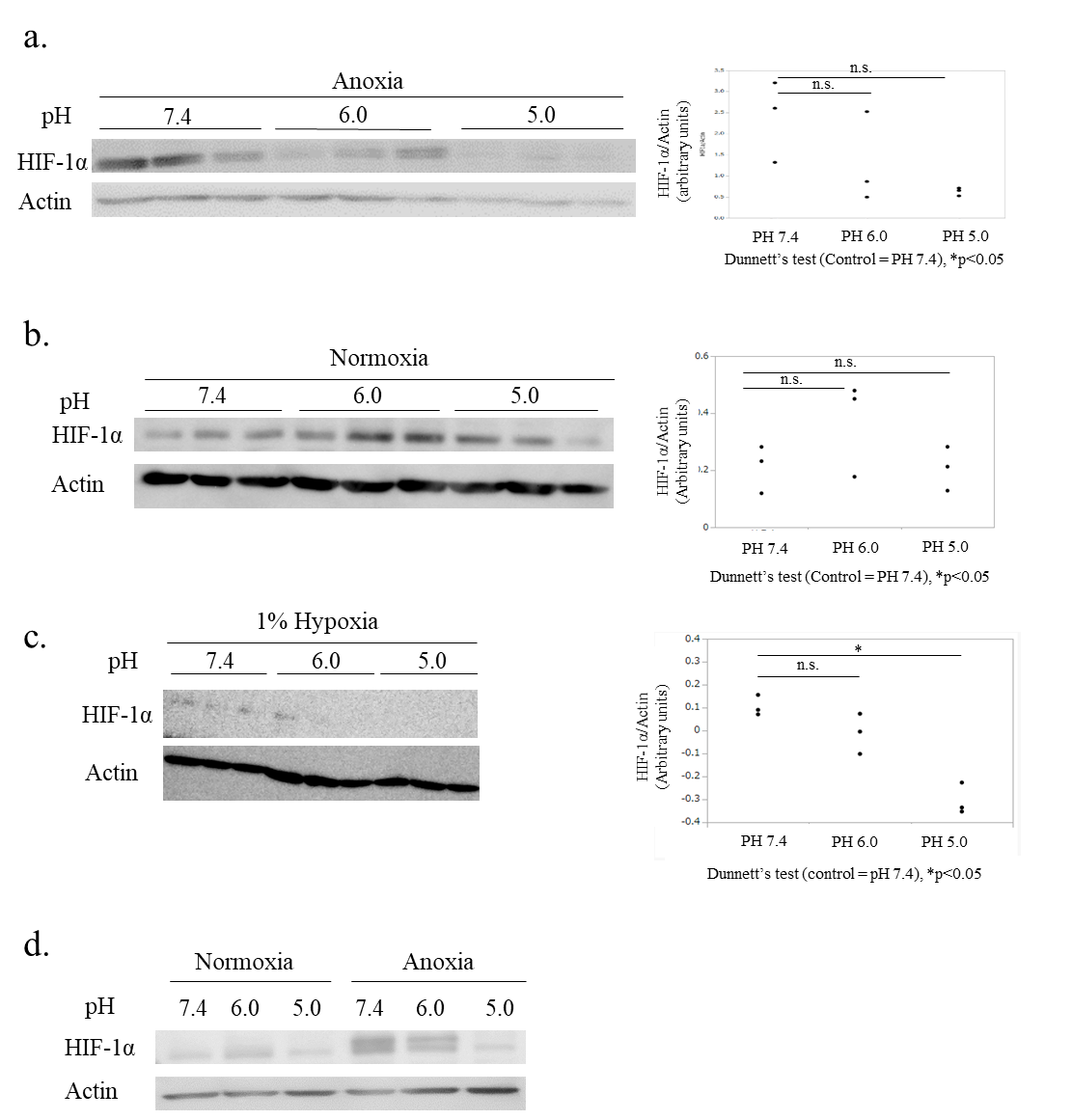


**Fig. S7: HIF-1α accumulation in different pH incubations under homogenous oxygen tension**

1. Quantitative analysis of HIF-1α protein in different pH incubations under homogenous oxygen tension using western blotting. According to quantitative analysis of HIF-1α bands normalized to those of actin, HIF-1α accumulation was suppressed at pH 5.0, even under anoxic conditions.
2. Quantitative analysis of HIF-1α protein in different pH incubations under normoxic conditions by western blotting. Quantitative analysis of HIF-1α bands normalized to those of actin did not show a significant change in pH 6.0 or pH 5.0 incubation, compared with incubation at pH 7.4.
3. Quantitative analysis of HIF-1α protein in different pH incubations under 1% O_2_ conditions by western blotting. HIF-1α bands normalized to those of actin decreased significantly at pH 5.0, compared with that at pH 7.4.
4. Representative figure of HIF-1α protein accumulation in different pH incubations under normoxic and anoxic conditions.


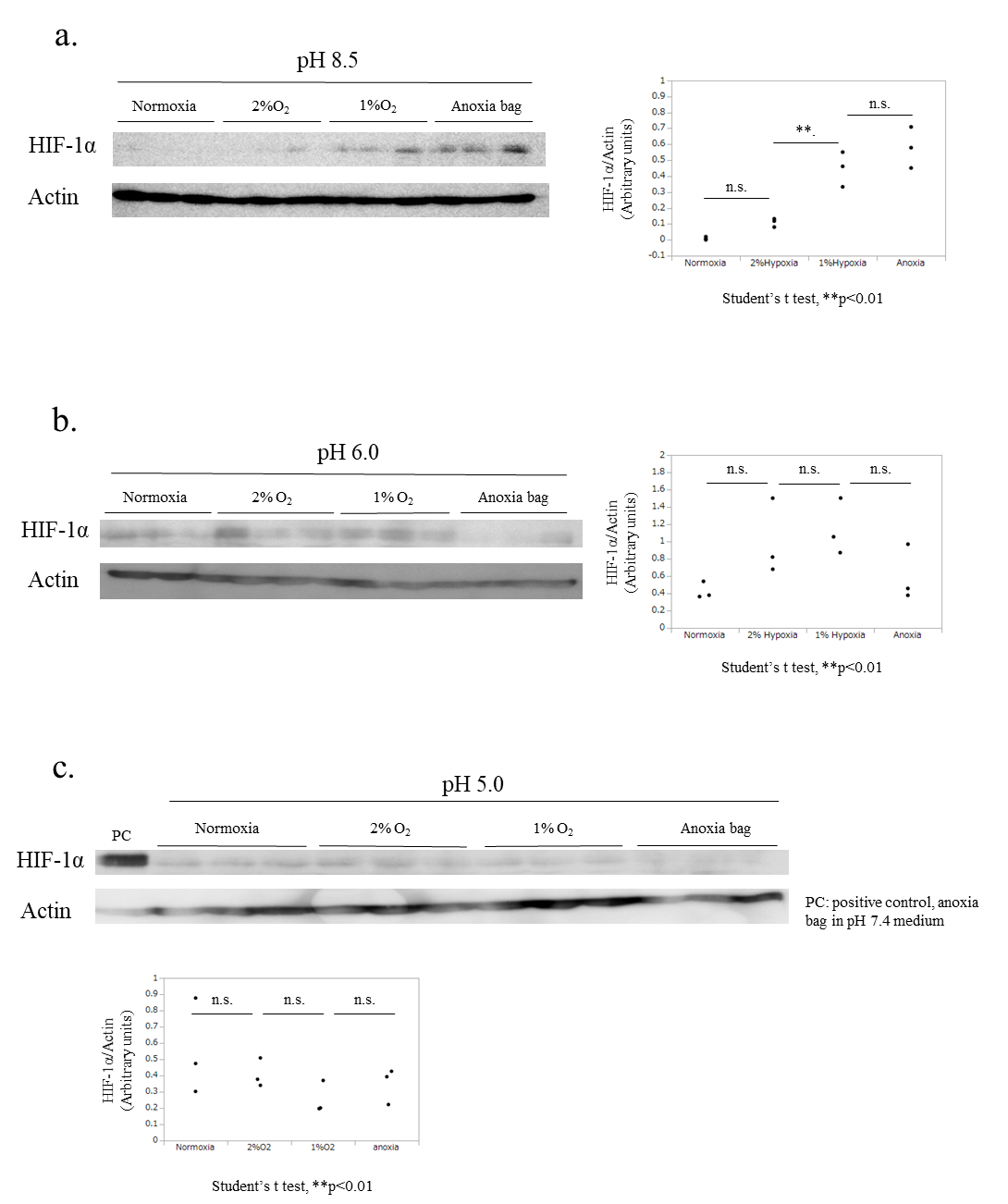


Fig.S8: **HIF-1α accumulation in different oxygen tensions under the same pH**

1. Quantitative analysis of HIF-1α protein in different oxygen tensions at pH 8.5 by western blotting. According to quantitative analysis of HIF-1α bands normalized to those of actin, HIF-1α accumulation increased under hypoxic conditions.
2. Quantitative analysis of HIF-1α protein in different oxygen tensions at pH 6.0 by western blotting. According to quantitative analysis of HIF-1α bands normalized to those of actin, HIF-1α accumulation did not show a significant change between normoxic and hypoxic conditions.
3. Quantitative analysis of HIF-1α protein in different oxygen tensions at pH 5.0 by western blotting. According to quantitative analysis of HIF-1α bands normalized to those of actin, HIF-1α accumulation did not show a significant change between normoxic and hypoxic conditions.


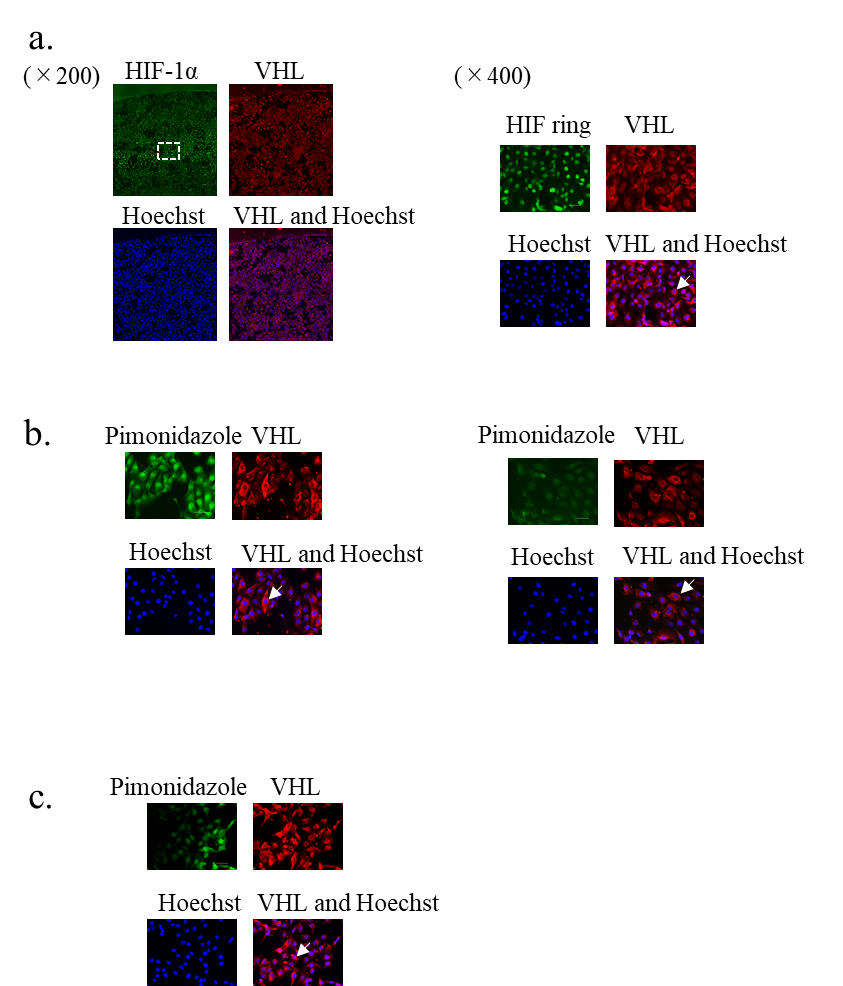


**Fig.S9: Immunocytochemistry of VHL in a coverslip model**

1. The ICC of VHL in a coverslip model incubated with pH 7.4 medium is shown in the upper images (x4). We studied VHL localization in the region of the HIF ring (dotted square) in a higher-power field (x400) (bottom images). VHL was localized in the cytosol, but not in the nuclei, over the whole area of a coverslip model with pH 7.4 incubation (arrow). Scale bars: 100 μm (×200) and 50 μm (×400)
2. ICC of VHL in a coverslip model incubated with pH 6.0 medium. ICC of VHL in both pimonidazole-positive (upper images) and negative (bottom images) areas showed that VHL was localized in the cytosol, but not in the nuclei (arrow). Scale bar: 50 μm
3. ICC of VHL in a coverslip model incubated with pH 5.0 medium. ICC of VHL in both pimonidazole-positive (right) and negative (left) areas showed that VHL was localized in the cytosol, but not in the nuclei (arrow). Scale bar: 50 μm


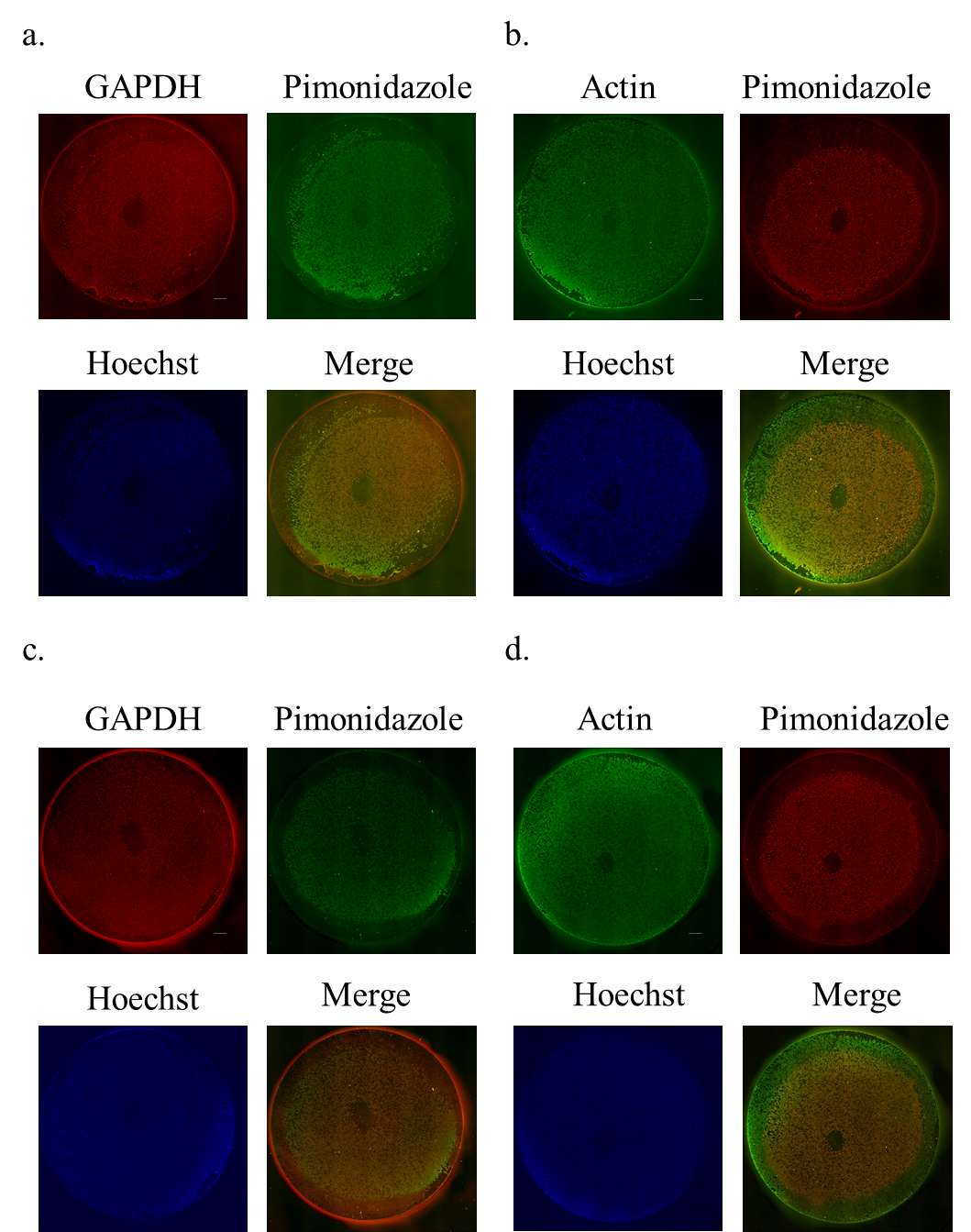


**Fig. S10: Immunocytochemistry with GAPDH and actin in acidic conditions in a coverslip model**

1. ICC of GAPDH under a coverslip model at pH 6.0. GAPDH was maintained in the whole area of the coverslip model. Scale bar: 1000 µm
2. ICC of actin under a coverslip model at pH 6.0. Actin was maintained in the whole area of the coverslip model. Scale bar: 1000 µm
3. ICC of GAPDH under a coverslip model at pH 5.0. GAPDH was maintained in the whole area of the coverslip model. Scale bar: 1000 µm
4. ICC of actin under a coverslip model at pH 5.0. Actin was maintained in the whole area of the coverslip model. Scale bar: 1000 µm


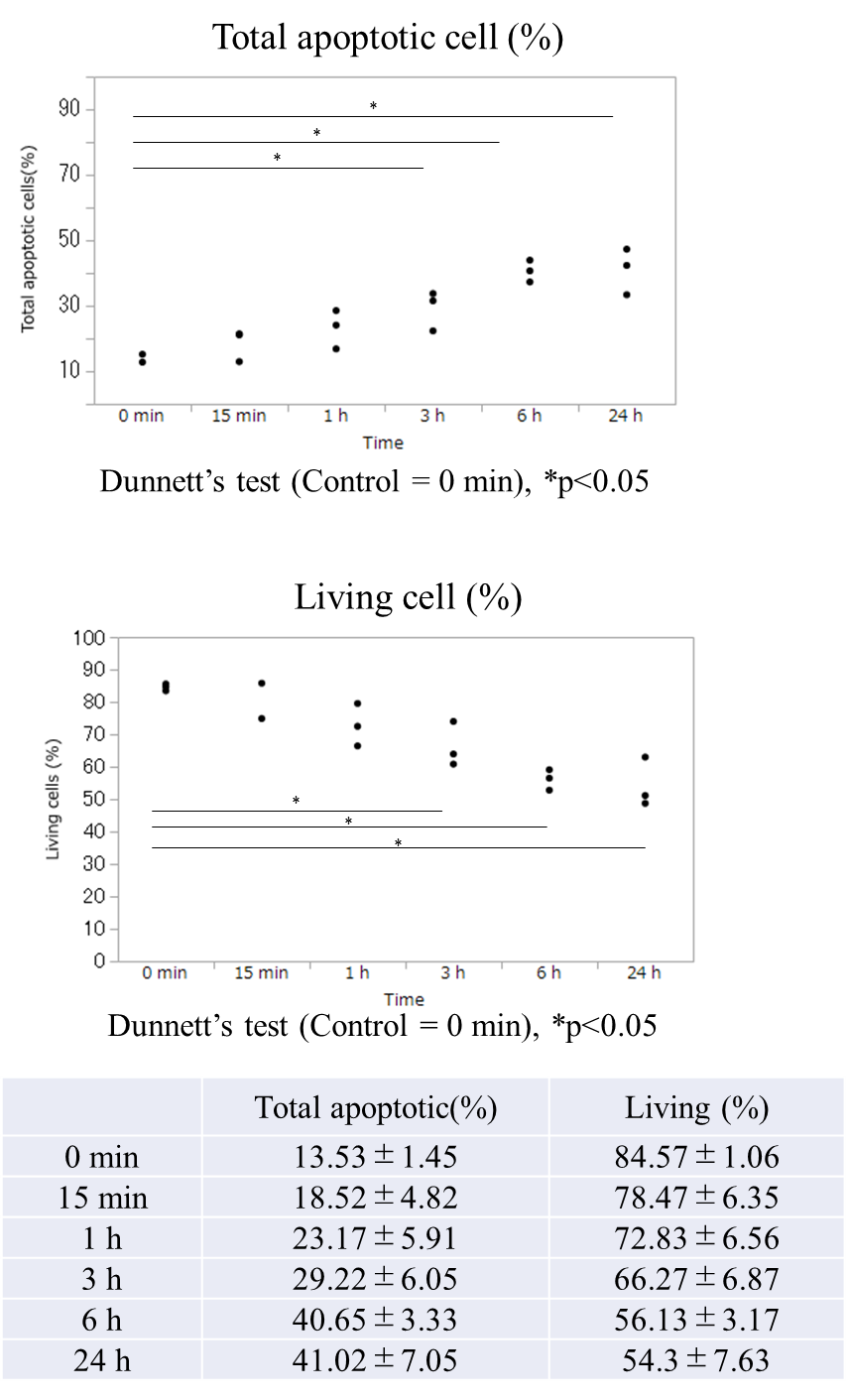


**Fig. S11: Cell viability in a coverslip model**

Evaluation of apoptotic cells under a coverslip by Muse cell analysis using Annexin V and Dead Cell Kits. The number of apoptotic cells increased over time after coverslip placement. Compared with control cells without a coverslip model, approximately 10 to 20% of HK-2 cells covered with a coverslip for 3 h became apoptotic. Each value was obtained from three independent experiments. Data are shown as mean ± SD.


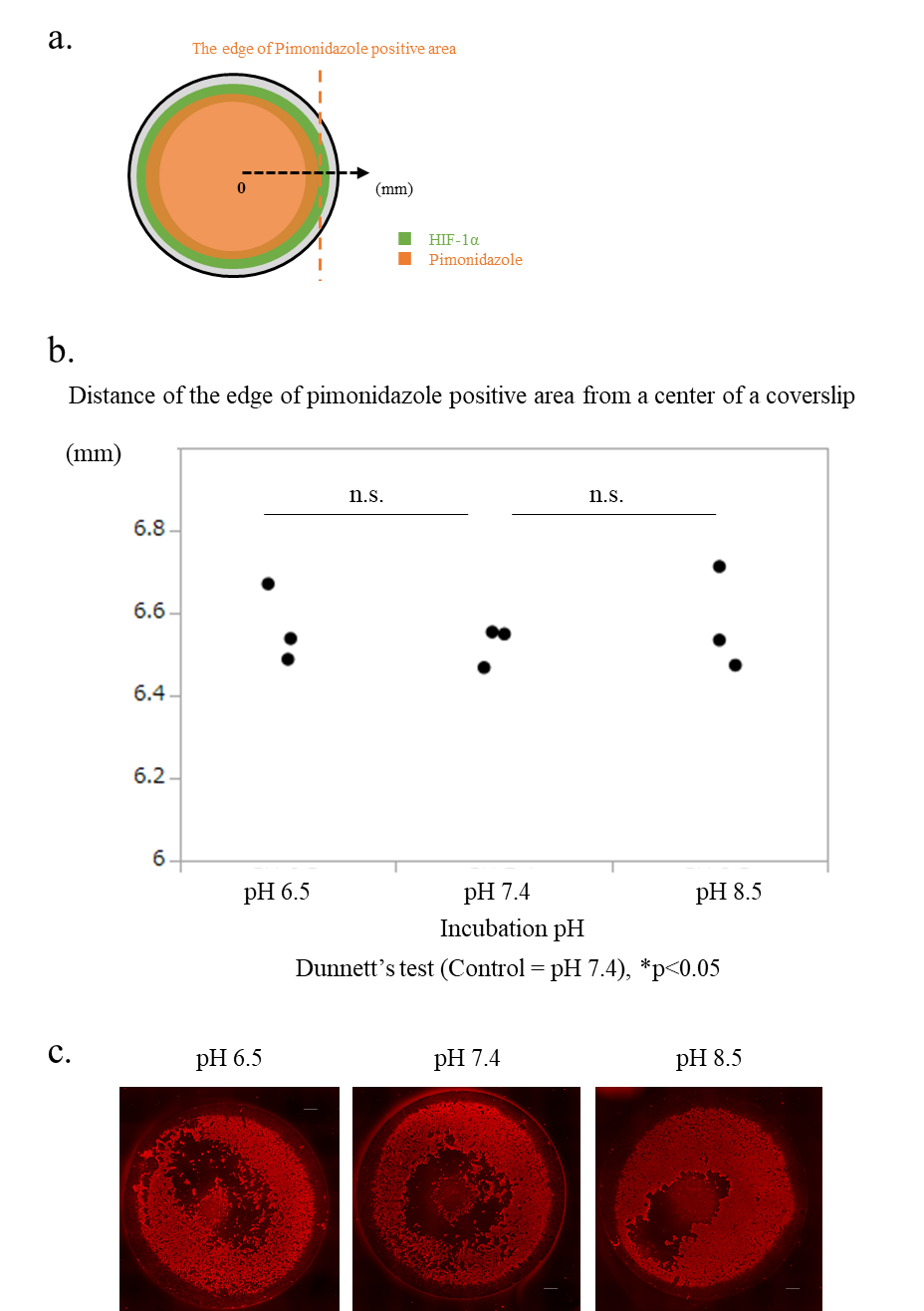


**Fig.S12: Effect of pH on a hypoxic marker, pimonidazole**

1. Diagram of the distance measurement of pimonidazole positive area from the center of a coverslip. The center of the coverslip was determined to be zero.
2. We compared the distance of the pimonidazole-positive area from the center of a coverslip at different pH levels in our coverslip model. The edge of the pimonidazole-positive area was comparable between pH 6.5, 7.4, and 8.5.
3. Representative images of pimonidazole-positive area at pH 6.5, pH 7.4, and pH 8.5 (from Fig.6c).

**Table S1: First antibodies used in the study**


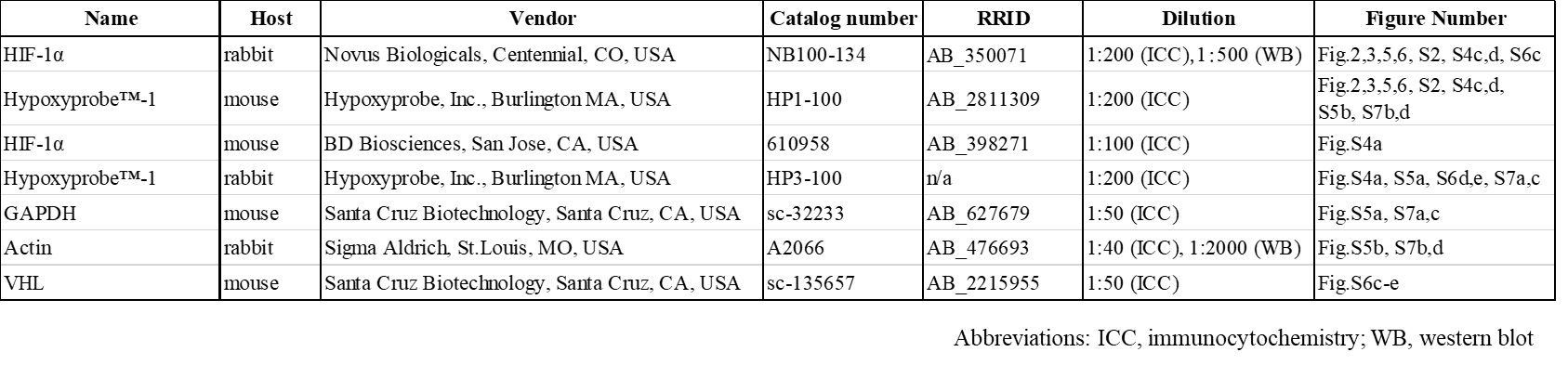


**Table S2: Correspondence between phosphorescence lifetime and the oxygen tension**


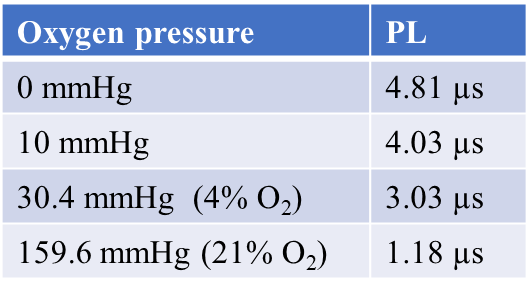

Supplement: Supplementary file 1 — Supplementary Material [file PHY2-9-e14689-s001.docx]
